# Proton NMR Enables the Absolute Quantification of Aqueous Metabolites and Lipid Classes in Unique Mouse Liver Samples

**DOI:** 10.3390/metabo10010009

**Published:** 2019-12-21

**Authors:** Aurélien Amiel, Marie Tremblay-Franco, Roselyne Gautier, Simon Ducheix, Alexandra Montagner, Arnaud Polizzi, Laurent Debrauwer, Hervé Guillou, Justine Bertrand-Michel, Cécile Canlet

**Affiliations:** 1 Toxalim-Research Centre in Food Toxicology, Toulouse University, INRAE UMR 1331, ENVT, INP-Purpan, Paul Sabatier University, F-31027 Toulouse, France; aurelien.amiel@yahoo.fr (A.A.); marie.tremblay-franco@inra.fr (M.T.-F.); roselyne.gautier@inra.fr (R.G.); simon.ducheix@univ-nantes.fr (S.D.); alexandra.montagner@inserm.fr (A.M.); arnaud.polizzi@inra.fr (A.P.); laurent.debrauwer@inra.fr (L.D.); herve.guillou@inra.fr (H.G.); 2 Metatoul-AXIOM platform, National Infrastructure for Metabolomics and Fluxomics, MetaboHUB, Toxalim, INRAE UMR 1331, F-31027 Toulouse, France; 3 Metatoul-Lipidomic Core Facility, MetaboHUB, I2MC U1048, INSERM, F-31432 Toulouse, France; justine.bertrand-michel@inserm.fr

**Keywords:** metabolomics quantitative profiling, lipidomics, ^1^H-NMR spectroscopy, liver, steatosis

## Abstract

Hepatic metabolites provide valuable information on the physiological state of an organism, and thus, they are monitored in many clinical situations. Typically, monitoring requires several analyses for each class of targeted metabolite, which is time consuming. The present study aimed to evaluate a proton nuclear magnetic resonance (^1^H-NMR) method for obtaining quantitative measurements of aqueous and lipidic metabolites. We optimized the extraction protocol, the standard samples, and the organic solvents for the absolute quantification of lipid species. To validate the method, we analyzed metabolic profiles in livers of mice fed three different diets. We compared our results with values obtained with conventional methods and found strong correlations. The ^1^H-NMR protocol enabled the absolute quantification of 29 aqueous metabolites and eight lipid classes. Results showed that mice fed a diet enriched in saturated fatty acids had higher levels of triglycerides, cholesterol ester, monounsaturated fatty acids, lactate, 3-hydroxy-butyrate, and alanine and lower levels of glucose, compared to mice fed a control diet. In conclusion, proton NMR provided a rapid overview of the main lipid classes (triglycerides, cholesterol, phospholipids, fatty acids) and the most abundant aqueous metabolites in liver.

## 1. Introduction

The liver is among the most metabolically diverse organs of the body, and it is involved in many metabolic processes. The liver plays a central physiological role in lipid metabolism; e.g., it hosts cholesterol synthesis, cholesterol degradation to bile acids, triglyceride production, and lipoprotein synthesis. The liver may be affected by many pathological aggressions. Associated with the obesity epidemic, Non Alcoholic Fatty Liver Diseases (NAFLD) is currently a major public health concern [1]. NAFLD ranges from benign fat accumulation to inflammatory steatohepatitis that may promote irreversible damage [1]. The current methods of diagnostic mostly rely on liver biopsies [2]. However, metabolomic approaches are extensively used for biomarker identification as well as for identification of metabolic pathways involved in the progression of lipid accumulation [3,4]. Therefore, there is a lot of interest in methods allowing the integration of both soluble metabolites and lipids from a single sample.

Metabolomics is currently established as a powerful investigation tool that provides rich information on metabolic disturbances in human disease. Mass spectrometry (MS) and nuclear magnetic resonance (NMR) are the two most widely used techniques in metabolomics. NMR spectroscopy has several advantages over MS, including high reproducibility, non-destructive analysis, a simple quantification approach, and minimal sample preparation [5]. ^1^H-NMR-based metabolomics is currently widely used to gain insights into liver disease mechanisms [6,7] or to evaluate drug hepatotoxicity [8,9] and environmental contaminants [10]. In most studies that used proton NMR-based metabolomics for liver samples, analyses have been performed on either aqueous extracts or lipidic extracts. Those analyses employed spectral binning, followed by multivariate statistical analyses to highlight changes in metabolite composition due to disease [11,12,13,14], alcohol consumption [15,16,17], or contaminant exposure [18]. 

Lipids are a diverse, ubiquitous group of compounds, which have many key biological functions. Many diseases alter lipid metabolism; thus, a better understanding of these pathologies can be gained by analyzing lipid composition. Due to the structural diversity among lipid molecules, lipidomic profiling is complex. For the least abundant lipids (i.e., free fatty acids, cholesterol, oxylipids), we typically choose powerful, targeted, but time-consuming approaches, like liquid chromatography (LC) or gas chromatography (GC) coupled to mass spectrometry (MS) techniques (i.e., LC-MS or GC-MS), which can provide absolute quantitative results, under certain conditions [19]. However, to evaluate the most abundant lipids (i.e., phospholipids, sphingolipids, and triacylglycerides), we typically use LC-MS approaches that are not targeted, even though they do not provide quantitative results [20]. NMR is an alternative method that provides rapid, and in particular, quantitative analyses of hepatic lipids. Some studies have analyzed intact liver samples (biopsies) with high-resolution magic angle spinning (HR-MAS) NMR spectroscopy to study metabolic disruptions in human chronic hepatitis and cirrhosis [21] or to study non-alcoholic fatty liver disease (NAFLD) in murine models [22]. Those studies were carried out directly in the solid tissues, without any extraction. With HR-MAS, both lipid and aqueous metabolites can be simultaneous observed in the same spectrum, but the resolution is low, and absolute quantification is complex, due to overlapping signals. Other studies have performed metabolic analyses on tissue extracts to study human hepatocellular carcinoma (HCC) associated with NAFLD or cirrhosis [23] or to study the progression from hepatic steatosis to nonalcoholic steatohepatitis (NASH) in mouse models [24]. NMR analyses of tissue extracts require sample processing and separate analyses for lipidic and aqueous extracts. However, liquid state NMR provides spectra with a better resolution than those recorded with the HRMAS technique, and absolute quantification can be performed when a standard is used at a known concentration. Only a few studies have reported the absolute quantification of lipidic metabolites in liver samples, based on ^1^H-NMR spectroscopy [24]. Indeed, lipid species contain many long-chain fatty acids, and therefore, many overlapping proton signals (e.g., (CH_2_)_n_ in fatty acids), which makes a detailed characterization of lipid species unfeasible. However, with ^1^H NMR spectroscopy, it is possible to identify and quantify different classes of lipids, such as cholesterol, triglycerides, phospholipids, mono-unsaturated fatty acids (MUFAs), and poly-unsaturated fatty acids (PUFAs). 

The present study aimed to evaluate ^1^H-NMR spectroscopy for the identification and absolute quantification of polar and non-polar metabolites in the same liver sample. First, we compared two extraction methods to optimize aqueous and lipidic metabolite extractions. Then, we optimized the absolute quantification of lipid species on mixtures of lipid standards, by comparing internal vs. external standards and various organic solvents. Finally, the optimized method was applied to investigate the effects of a diet deficient in essential fatty acids on liver mouse metabolism. Hepatic lipids were quantified with both ^1^H-NMR spectroscopy and conventional methods to compare the results and validate the methodology. 

## 2. Results

### 2.1. Comparison of Extraction Methods 

Sample preparation represents a crucial step in metabolomic studies. In this study, we aimed to obtain the best preparation for both polar and lipid molecules. Several classical solvent systems have been developed for liver extractions [25]. Among the various possibilities, we chose Bligh and Dyer [26] and Folch [27] extractions, because these two methods resulted in a biphasic solvent system. Moreover, these were the main extraction methods used in our lab for targeted lipidomics, performed with conventional methods, and for NMR-based metabolomics [28]. With these extraction methods, the upper phase contained the polar (aqueous) fraction, and the bottom phase contained the lipids. When we tested the Bligh and Dyer extraction method with 50 mg of liver, we noticed the presence of an emulsion, which made it difficult to separate the aqueous and organic phases. With the Folch extraction method, the organic phase was washed with a saturated NaCl solution, and the phases were well separated.

**Figure 1:**
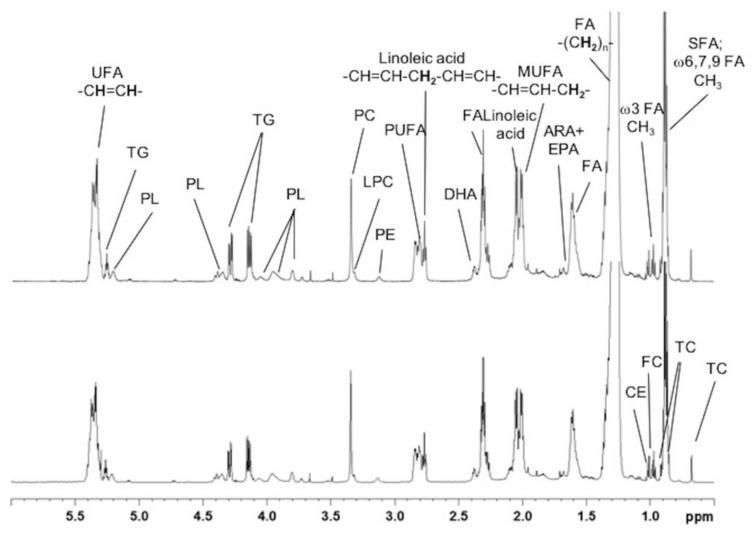
600 MHz ^1^H-NMR spectra of lipophilic extracts from mouse liver samples. The Folch extraction method (top) and the Bligh and Dyer extraction method (bottom) show the same peaks. The peaks are labeled in only one panel for clarity, as follows: FC, free cholesterol; CE, cholesterol ester; TC, total cholesterol; FA, fatty acids; SFA, saturated fatty acids; ARA, arachidonic acid; EPA, eicosapentaenoic acid; MUFA, monounsaturated fatty acids; DHA, docosahexaenoic acid; PUFA, polyunsaturated fatty acid; PE, phosphatidylethanolamine; LPC, lysophosphatidylcholine; PC, phosphatidylcholine; PL, phospholipids; TG, triglycerides; UFA, unsaturated fatty acids.

**Figure 2:**
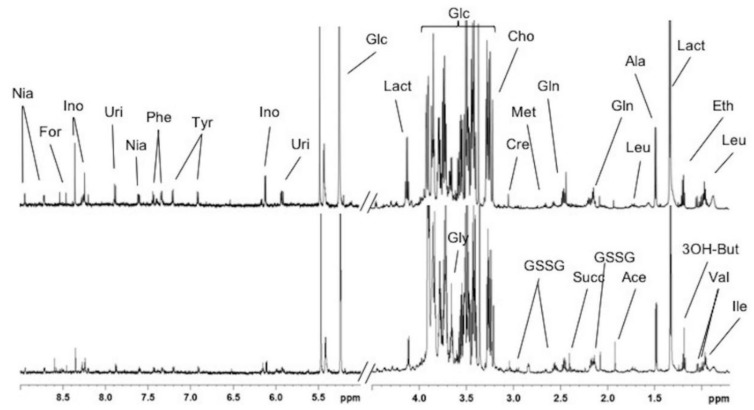
600 MHz ^1^H-NMR spectra of aqueous extracts from mouse liver samples. The Folch extraction method (top) revealed more peaks than the Bligh and Dyer extraction method (bottom). The peaks are labeled in only one panel for clarity, as follows: Ile, isoleucine; Val, valine; 3OH-But, 3-hydroxybutyrate; Ace, acetate; GSSG, glutathione oxidized; Succ, succinate; Gly, glycine; Leu, leucine; Eth, ethanol; Lact, lactate; Ala, alanine; Gln, glutamine; Met, methionine; Cre, creatine; Cho, choline; Glc, glucose; Uri, uridine; Ino, inosine; Tyr, tyrosine; Phe, phenylalanine; Nia, niacinamide; For, formate.

A visual assessment of the ^1^H-NMR spectra revealed that the two extraction procedures produced similar peak coverages and intensities for the extracted lipids (Figure 1). For the aqueous extracts, the intensities were higher in the aromatic region (δ 9.0–6.0 ppm) with the Folch extraction method than with the Bligh and Dyer method (Figure 2). 

For each method, the buckets representing metabolites with signal-to-noise ratios above 10 (SNR > 10) were annotated by comparing the chemical shifts in the 1D ^1^H-NMR spectra with those of reference spectra recorded under the same conditions and reference spectra deposited in the Biological Magnetic Resonance Databank [29] and the Human Metabolome Database [30]. We could identify 29 metabolites present in the polar fraction and eight lipid classes present in the non-polar fraction. ^1^H-NMR resonance assignments of aqueous and lipidic metabolites are shown in Tables S1 and S2, respectively, with the chemical shifts, multiplicity, and coupling constants of the signals elucidated in the ^1^H-NMR spectra for both the water- and lipid-soluble extracts from mouse liver. For the lipidic extracts, among the selected 52 buckets, 50 were detected with an SNR > 10 with both extraction methods, which suggested that there was no significant difference between the two extraction methods, based on this criterion. For the aqueous extracts, among the selected 80 buckets, 40 and 58 were detected with an SNR > 10 with the Bligh and Dyer and the Folch extraction methods, respectively. This finding confirmed that the Folch method provided better extraction of the aqueous metabolites.

**Figure 3:**
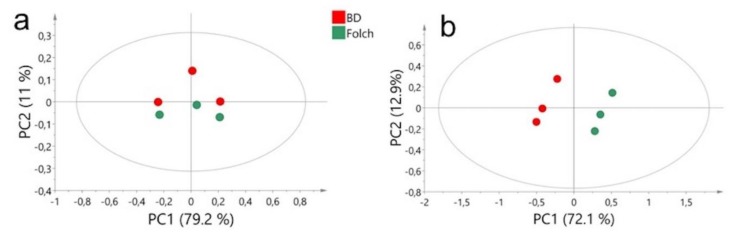
Principal component analysis score plot of the two extraction methods. (**a**) Liver lipidic extracts (*n* = 3); (**b**) liver aqueous extracts (*n* = 3). BD = Bligh and Dyer extraction method (red); Folch = Folch extraction method (green). Ellipses indicate the 95% confidence region.

**Figure 4:**
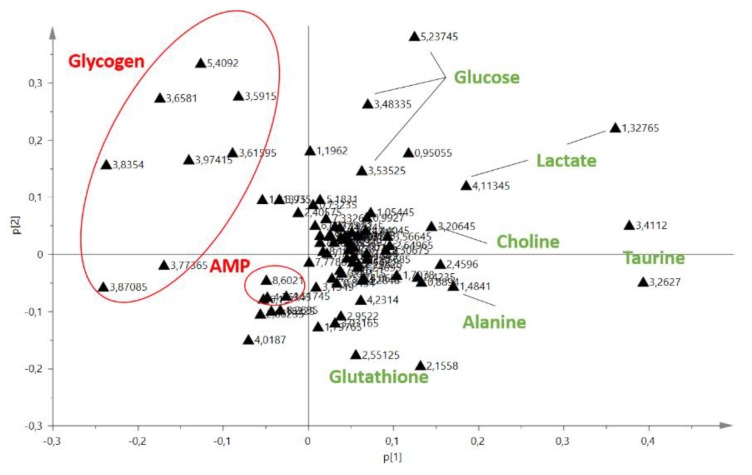
Principal component analysis loading plot of the two extraction methods for aqueous liver extracts. NMR buckets with the highest absolute loadings contributed the most to the separation between the two extraction methods and were annotated. Metabolites in red were elevated in Blye and Dyer extraction and metabolites in green were elevated in Folch extraction.

We performed a multivariate analysis combined with a principal component analysis (PCA) of the NMR buckets that described components of the extracts from both methods. We found that, for lipidic extracts, the two extraction methods were separated along the second principal component, which explained 11% of the variability. For aqueous extracts, the samples were separated along the first principal component, which explained 72.1% of the variability. These results suggested that the aqueous metabolites extracted were significantly different between these two extraction methods (Figure 3). The loading plot for aqueous liver extracts showed signals that contributed to the separation between extraction methods (Figure 4). Taurine, lactate, glucose, choline, alanine, glutathione and others amino acids are elevated in Folch extraction, and glycogen and adenosinemonophosphate (AMP) are elevated in Blye and Dyer extraction.

Generally, when the two classical Bligh and Dyer and Folch lipid extraction methods were used, most studies focused either on the analysis of lipid extracts using MS techniques [31] or on the analysis of aqueous extracts by NMR spectroscopy [32,33], but few studied reported the analysis of both extracts. Our results showed that, in this case, the Folch extraction method was more efficient in extracting aqueous metabolites than the Bligh and Dyer method, but the two extraction methods showed no differences in extracting lipidic metabolites. The variability was similar with both extraction methods. Therefore, we selected the Folch extraction method for all subsequent analyses in this study.

### 2.2. Absolute Quantification of Lipidic and Aqueous Metabolites

In NMR, absolute quantification requires the use of an internal or external standard at a known concentration. A number of reference compounds are available for quantitative NMR analysis. The most widely used reference compounds for chemical shift referencing and quantitative analysis are tetramethylsilane (TMS, organic solubility), 3-(trimethylsilyl)-1-propane sulfonic acid sodium salt (DSS, aqueous solubility), and 3-(trimethylsilyl)propionic acid sodium salt (TSP, aqueous solubility) [34]. In this study, for aqueous extracts, we used TSP as an internal standard, TSP was directly dissolved in the sample. Signals used for absolute quantification of aqueous metabolites were indicated in bold in Table S1. For lipid quantifications, TMS was directly dissolved in the sample as an internal standard; as an external standard, TSP was dissolved in deuterated water (D_2_O) in a coaxial capillary tube for mixtures of lipid standards. The final solvent in an NMR experiment is another key point: in the literature, deuterated chloroform (CDCl_3_) and a mixture of CDCl_3_ and deuterated methanol (CD_3_OD) are mostly used for lipidomic analyses. In the present study, we tested both pure deuterated chloroform (CDCl_3_) and a 4:1 (*v/v*) mixture of CDCl_3_ and CD_3_OD for the final solvent. The lipid extracts and standards were dissolved in these solvents.

The metabolites were quantified according to the following expression:

(1) $$Cx=\frac{\frac{Ix\times Cs}{Nx}}{\frac{Is}{Ns}}\times \frac{V}{M}$$

where *Cx* is the metabolite concentration, *Ix* is the integral of the metabolite proton peak, *Nx* is the number of protons in the metabolite proton peak, *Cs* is the standard concentration, *Is* is the integral of the standard proton peak, *Ns* is the number of protons in the standard proton peak, *V* is the volume of the analyzed extract, and *M* is the weight of liver tissue analyzed.

The quantification of lipid species was simple for isolated peaks without any signal overlapping, such as the total cholesterol (TC, singlet, 3H, 0.68 ppm), ω-3 fatty acids (ω-3 FAs, triplet, 3H, 0.97 ppm), arachidonic and eicosapentaenoic acids (ARA+EPA, multiplet, 2H, 1.68 ppm), MUFAs (multiplet, 4H, 2.01 ppm), docosahexaenoic acid (DHA, multiplet, 4H, 2.38 ppm), linoleic acid (triplet, 2H, 2.27 ppm), phosphatidylethanolamine (PE, multiplet, 2H, 3.12 ppm), phosphatidylcholine and lysophosphatidylcholine (PC+LPC, singlet, 9H, 3.20 ppm), triglycerides (doublet of doublets, 2H, 4.29 ppm), and sphingomyelin (SM, multiplet, 1H, 5.70 ppm). Free cholesterol (FC) and cholesterol ester (CE) could be quantified based on the signals at 1.01 and 1.02 ppm, respectively, with the deconvolution algorithm available in Topspin software (Bruker, Rheinstetten, Germany). PUFAs could not be quantified directly, because the number of protons that corresponded to the signals at 2.82 ppm could not be determined precisely. As previously mentioned by Vidal et al. [35], it was possible to determine the molar percentage of unsaturated fatty acids (UFA) with the following equation:

(2) $$UFA\;\left(\%\right)=\;\frac{100\times \left(\left(2\times A7\right)+A9\right))}{\left(\left(2\times A9\right)+\left(4\times A8\right)\right)}$$

where *A*7 is the signal integration between 1.92 and 2.15 ppm corresponding to the functional group –C**H_2_**-CH=CH- acyl group except for –CH_2_- of DHA acyl group; *A*8 is the signal integration between 2.25 and 2.36 ppm corresponding to the functional group –OCO-C**H_2_**- acyl group except for DHA; and *A*9 is the signal integration between 2.36 and 2.42 ppm corresponding to the functional group –OCO-C**H_2_**-C**H_2_**- of DHA acyl group.

The total fatty acid concentration could be determined as the sum of the signals at 0.97 ppm (ω-3 FAs) and 0.88 ppm. From this value, we could calculate the concentrations of UFAs, saturated fatty acids (SFAs), and PUFAs.

To validate our quantification method, we analyzed five mixtures of five lipid standards (triglycerides C17:0; FC; oleate cholesterol; DHA; and linoleic acid) and five mixtures of three phospholipid standards (PC, PE, and SM) at different concentrations (Table S3). These 10 mixtures represented most of the common signals in ^1^H NMR spectra of lipophilic extracts from liver samples. Annotated NMR spectra of mixture of lipid standards (mix3) and mixture of phospholipid standards (mix6) are presented in Figures S1 and S2 respectively. The concentrations of lipid species were determined by calibrating to the internal and external standards in the two organic solvents. Table 1 and Table 2 list the correlations between the concentrations obtained with NMR signal integration and their respective real concentrations, obtained by internal or external standard calibrations. The corresponding scatter plots and linear regressions are available in Figures S3 and S4 (TSP and CDCl_3_), Figures S5 and S6 (TMS and CDCl_3_), Figures S7 and S8 (TSP and CDCl_3_-CD_3_OD mixture), and Figures S9 and S10 (TMS and CDCl_3_-CD_3_OD mixture). All hypotheses underlying the linear regression (linearity, independence, homogeneity) were validated by the residue analysis. The results showed that the external standard and the solvent mixture CDCl_3_/CD_3_OD (4:1) provided the best correlations (r > 0.9) and linearity in the regression analyses (i.e., slopes that approximated 1). 

These results also suggested that TMS was unsuitable for quantitative analyses and that the nature of the solvent was important. Phospholipids contain polar headgroups and nonpolar fatty acyl residues, which lead to line broadening in ^1^H-NMR spectroscopy; thus, the quantification was not accurate. Accordingly, we used an external standard and the solvent mixture, CDCl_3_/CD_3_OD (4:1 v/v), for the biological study.

### 2.3. Analytical Validation with Liver Samples in a Dietary Intervention Study

We evaluated the use of ^1^H-NMR spectroscopy for quantifying aqueous and lipidic metabolites in liver samples from mice fed the following diets: COCO (deficient in essential fatty acids, with 5% saturated FA-rich oil), REF (balanced diet with 5% REF oil), and FISH (n-3 PUFA-enriched diet, with 5% PUFA-rich oil). Lipids were quantified with ^1^H-NMR spectroscopy and an external standard dissolved in the mixture of solvents, CDCl_3_/CD_3_OD (4:1 *v/v*). 

#### 2.3.1. Comparison with GC-FID Data

We compared ^1^H-NMR spectroscopy results to those obtained with GC with a flame ionization detector (GC-FID). We took the GC-FID data as the reference method for quantifying fatty acids and neutral lipids. Table 3 shows the correlation between NMR quantifications (expressed in nmol/mg of liver) and GC-FID quantifications. The correlations were good for all tested lipids (r > 0.8, *p* < 0.01), except for ARA+EPA.

#### 2.3.2. Comparison with LC-MS Data

The quantification of phospholipids with LC-MS provided relative values, due to the unavailability of standards for calibrating LC-MS quantifications. Thus, for phospholipids, we could only compare relative differences between diets. Table 4 shows the ratios of phospholipid concentrations between the test and reference diets. These ratios were calculated with results from the ^1^H-NMR and LC-MS analyses to compare the two methods. The relative error between LC-MS and NMR values were smaller than 6% for PE, PC+LPC and total PC except for SM (10%). We can assume that the quantification ratios provided by the ^1^H-NMR analyses were similar to the quantification ratios provided by the LC-MS analyses. For SM ratio, the relative error was larger because the NMR signal was weak.

#### 2.3.3. Comparison with LipSpin Results

LipSpin is a bioinformatic tool for the automatic quantification of lipid species, which uses ^1^H- NMR spectra of biological matrices [36]. Lipid quantifications rely on line-shape fitting analyses of spectral regions, from which individual signal areas are obtained. The user can select a signal pattern optimized for blood serum, which is provided by the algorithm; however, we designed our own signal pattern and imported our standard spectra, because our experimental conditions were different (i.e., solvents and acquisition parameters). This tool is user-friendly, fast, and requires only the import of NMR spectra. LipSpin provided the integration values for SFA, ω-3 FA, MUFA, ARA+EPA, linoleic acid, FC, EC, triglyceride, PL, PE, SM, PC, and LPC signals. The concentration was calculated for each lipid species based on the total cholesterol concentration determined with GC-FID. Table 5 shows the correlations between LipSpin quantifications (expressed in nmol/mg liver) and our NMR quantifications. We found good correlations for ω-3 FA, MUFA, linoleic acid, EC, and triglycerides (r > 0.8, *p* < 0.01), but low correlation values were obtained for other lipid species.

#### 2.3.4. Biological Results

**Figure 5:**
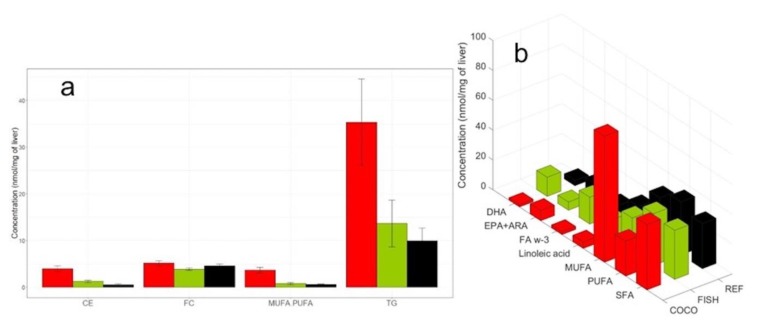
The quality of dietary fatty acids (FAs) affected hepatic lipid composition. Mouse liver lipid compositions were measured after 12-week diets of COCO (red) or FISH (green), compared to a reference (black) diet as revealed by ^1^H-NMR spectra. (**a**) Liver cholesterol (CE and FC) contents, triglyceride (TG) contents, and MUFA/PUFA ratios. (**b**) Liver fatty acid contents. Abbreviations: COCO, a diet with 5% saturated FA-rich oil; REF, a diet with 5% reference oil; FISH, a diet with 5% n-3 long-chain PUFA-rich oil; CE, cholesterol ester; FC, free cholesterol; MUFA, monounsaturated FAs; PUFA, polyunsaturated FAs; TG, triglycerides; DHA, docosahexaenoic acid; EPA, eicosapentaenoic acid; ARA, arachidonic acid; FA w-3, omega-3 FAs; SFA, saturated FAs.

Figure 5 shows the mean concentrations of lipid species in the livers of mice fed each diet obtained from ^1^H-NMR spectra. Lipid modifications were in good agreement with the results previously obtained with the standard GC-FID method [37]. The ^1^H-NMR and GC-FID methods also showed comparable significant increases in triglycerides and CE in mice fed the COCO diet compared to mice fed the REF diet. In addition, the ^1^H-NMR method highlighted significant increases in fatty acids, MUFAs, and SFAs, and significant decreases in PUFAs (DHA, EPA, linoleic acid, and ω-3 FA) in mice fed the COCO diet compared to mice fed the REF diet. Moreover, we observed significant increases in DHA and ω-3 FAs in mice fed the FISH diet compared to mice fed the REF diet. Although we did not compare the results on aqueous metabolites with results obtained with other methods, we found no significant changes in aqueous metabolites among the three diets, based on absolute concentrations.

Absolute quantification of metabolites (i.e., both aqueous and lipidic metabolites) requires knowing the number of protons under each signal, which is given after all NMR signals are identified. Thus, it is important to work with well-resolved signals with no overlap to ensure the integration of pure signals. Because it was difficult to identify all the signals in a NMR spectrum of a biological sample, due to numerous potential line overlaps, we applied the binning method to NMR data, so as to use the entire spectrum to compare metabolic profiles between the three groups of animals.

**Figure 6:**
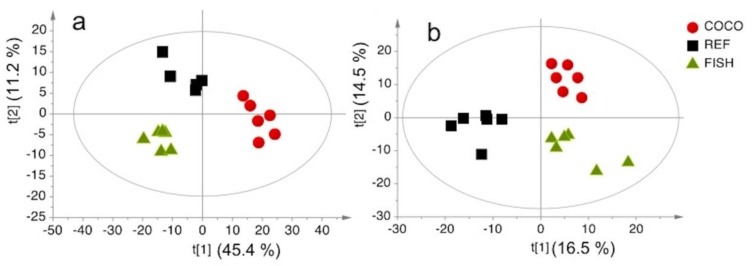
Partial Least Squares-Discriminant Analysis score plot for ^1^H-NMR data. (**a**) Liver lipidic extracts (R^2^ = 98.5%; Q^2^ = 0.884); (**b**) liver aqueous extracts (orthogonal signal correction filtration, 53.4% of the remaining variability, R^2^ = 98.7%; Q^2^ = 0.883). Ellipses indicate the 95% confidence regions. Abbreviations: COCO, a diet with 5% saturated FA-rich oil; REF, a diet with 5% reference oil; FISH, a diet with 5% n-3 long-chain PUFA-rich oil.

To compare the metabolic profiles of livers from mice fed different diets, we performed a partial least squares-discriminant analysis (PLS-DA), based on the ^1^H-NMR spectra. Mice were fed COCO, REF, and FISH diets. For the lipidic extracts, mice fed the COCO diet were well separated on the first component from mice fed the REF and FISH diets (Figure 6a). This component could explain 47.7% of the variability. In addition, mice fed the FISH diet were separated from mice fed the REF diet on the second component, which explained 38.9% of the variability. For aqueous extracts, prior to PLS-DA modeling, we applied an orthogonal signal correction to the data to filter out variations that were unrelated to the diet. Figure 6b shows that mice fed the COCO or FISH diets were clearly separated from mice fed the REF diet on the first component, which explained 42.1% of the variability. We found eight lipid species and fifteen aqueous metabolites that were responsible for the separation between diets (Table 6). From binning data, we observed significant increases in triglyceride, and MUFA levels in mice fed the COCO diet compared to mice fed the REF diet. We also observed changes in aqueous metabolites between the COCO and REF diets. Compared to the REF diet, the COCO diet caused significant increases in 3-hydroxybutyrate, alanine, glycerophosphocholine, inosine, lactate, leucine, phenylalanine, succinate, threonine, tyrosine, and valine and a significant decrease in glucose (Table 6).

## 3. Discussion

### 3.1. Lipid Quantification: Comparison to Other Methods

In this study, we evaluated the usefulness of an NMR method for quantifying lipid species, based on peak integration and a linear combination of integrals with a standard. We started by optimizing the standard (either internal or external), the organic solvent in which the lipid species would be dissolved, and a mixture of standard lipids. We found that an external standard, TSP, dissolved in D_2_O in a coaxial tube provided better results than the internal standard, which was TMS dissolved in the sample. Because TMS is a volatile compound, it was difficult to determine its exact concentration after completing the sample preparation process. With an external standard, quantification was more accurate, because there was no interaction between the standard solution in the coaxial tube and the molecules in the sample. In previous studies, several organic solvents were used to analyze lipidic extracts with NMR, including CDCl_3_ alone [38] or mixtures of solvents, such as CDCl_3_/CD_3_OD [17] or CDCl_3_/CD_3_OD/D_2_O-EDTA [39]. Because phospholipids contain polar headgroups and nonpolar fatty acyl residues, they form bilayers in an aqueous environment and ‘inverse’ micelles in an organic solvent, which are characterized by line broadening in ^1^H-NMR spectroscopy. When a solvent mixture contains both nonpolar and polar solvents, stable micelles are formed, which results in well resolved NMR signals and more accurate quantification [39]. 

The NMR method developed here for lipid quantification provided good correlations with the quantifications of different lipid classes determined with GC-FID and LC-MS. Both GC-FID and LC-MS are time-consuming, because they require an extraction and analysis method per class of lipids, as well as calibration curves for absolute quantification. In ^1^H-NMR spectroscopy, deconvolution or line fitting is useful for overlapping signals. We compared our ^1^H-NMR spectroscopy method with the LipSpin method for the quantification of lipid species [36]. The LipSpin tool is user-friendly, fast, requires only the import of NMR spectra, and it automatically provides the integration value for each lipid species. We obtained good correlations between our method and the LipSpin method for ω-3 FA, MUFA, linoleic acid, EC, and triglycerides. For the other lipid species, the correlations between quantification values obtained with the two methods were not very good, likely due to the lower resolution of our spectra; indeed, some peaks, like PC, LPC, and SM, were not well resolved. In addition, we had not optimized all the parameters available in the LipSpin tool.

### 3.2. Metabolic Differences between Livers of Mice Fed an Essential Fatty Acid-Deficient Diet or a Control Diet

In this study, we investigated the metabolic disturbances that occurred during the development of steatosis induced by an essential PUFA deficiency. Deficiencies in essential fatty acids are well-known to promote de novo lipogenesis through transcriptional processes [40] involving the transcription factors, SREBP1c [41], ChREBP [42], and LXR [37]. These processes increase the expression and activity of hepatic enzymes involved in de novo lipogenesis (ACC, FAS), elongation (ELOVL6), and desaturation (SCD1). In turn, the elevated activities of these enzymes cause an increase in triglycerides enriched in MUFAs, such as oleic acid (C18:1n-9). Our metabolomics approach revealed that, with the COCO diet, liver triglycerides and MUFAs were up-regulated, as expected, but we also observed up-regulated levels of liver CEs, 3-hydroxybutyrate, alanine, glycerophosphocholine, inosine, lactate, leucine, phenylalanine, succinate, threonine, tyrosine, and valine, compared to mice fed the REF diet. In addition, we observed down-regulated levels of phospholipids and glucose in livers of mice fed the COCO diet compared to mice fed the REF diet. A similar increase in CE was previously reported in lipidic extracts of livers from mice with NASH [43]. That study also highlighted a reduced PUFA to MUFA ratio in NASH, but they observed no difference in triglyceride levels, compared to healthy mice. A reduced PUFA/MUFA ratio is considered a marker of lipid peroxidation in association with oxidative stress. In cancer tissues, such as HCC, a glycolytic shift was observed, with high levels of lactate and low levels of glucose [23,44]. Liver mitochondria produce 3-hydroxybutyrate during fatty acid oxidation. High levels of 3-hydroxybutyrate were also found in human HCC tissues. The aromatic amino acids, phenylalanine and tyrosine, are oxidized in the TCA cycle, after conversion into fumarate. Therefore, accumulations of aromatic amino acids and 3-hydroxybutyrate suggested that mitochondrial function and inflammatory status were impaired in the livers of mice fed the COCO diet. 

The present study also revealed changes in amino acid metabolism associated with the COCO diet. Previous studies have shown that changes in branched chain amino acid levels in human liver occurred in hepatic pathologies, such as NASH or alcohol-related liver damage [45]. We observed elevated hepatic concentrations of leucine, and valine, consistent with previous reports that hepatic amino acid metabolism was abnormally regulated, and branched chain amino acid oxidation was reduced in these pathologies. Elevations in alanine were also described in hepatic pathologies [6]. 

### 3.3. Advantages and Limitations of ^1^H-NMR Spectroscopy for Metabolic Profiling in Liver

We evaluated a simple protocol for the simultaneous characterization of lipidic and aqueous metabolic profiles in mouse liver tissues. We used NMR spectroscopy to identify and quantify both polar and non-polar metabolites. ^1^H-NMR spectroscopy is often used to quantify aqueous metabolites in metabolomic studies, but it has rarely been used for quantifying lipid species. Currently, there is much interest in using this technique for obtaining absolute quantifications of complex lipids, such as phospholipids or sphingolipids, which cannot be obtained with MS. Furthermore, NMR is a non-destructive technique; therefore, the analyzed sample can subsequently be used in MS analyses, which can provide a molecular species characterization for each family. ^1^H NMR has some drawbacks, such as low sensitivity, signal overlapping, and low resonance discrimination. Nevertheless, this technique can provide a rapid quantitative overview of the major lipid classes (fatty acids, triglycerides, phospholipids, and cholesterol) with a simple, single extraction, without extensive sample preparation, and due to its spectral linearity, without the need of multiple internal standards for quantitative estimations.

## 4. Materials and Methods 

### 4.1. Animals

In vivo studies were conducted under E.U. guidelines for the use and care of laboratory animals, and they were approved by an independent Ethics Committee (TOXCOM/0043/NL AP). To address the relative contribution of the quantity and quality of dietary FAs to triglyceride accumulation, we fed 6-week old male C57BL6 mice (Charles River, Les Oncins, France) different diets for 12 weeks (*n* = 6 in each group). The diets were as follows: one contained 5% saturated FA-rich oil (COCO), a second contained 5% reference oil (REF), and a third contained 5% n-3 long-chain PUFA-rich oil (FISH) [37]. At sacrifice, the liver was collected and immediately cut into samples that were snap-frozen in liquid nitrogen and stored at −80 °C until use for NMR and GC analysis.

### 4.2. Extraction Procedure

Liver samples (100–120 mg) were homogenized in 1.2 mL methanol in the Fastprep-24 homogenizer (MP Biomedicals, Irvine, CA, USA). For the comparison of extraction methods, a homogenate that corresponded to 50 mg of tissue was extracted, with modifications, according to the method described by Bligh and Dyer [26], in dichloromethane/methanol/water (2.5:2.5:2.1, *v/v/v*), and a second homogenate from the same sample that corresponded to 50 mg of tissue was extracted according to the method described by Folch [27] in dichloromethane/ methanol/NaCl 0.9% in water (2:1:0.2, *v/v/v*). After centrifugation (1000× *g*, 15 min, 4 °C), the solutions separated into an upper methanol/water phase (with polar metabolites) and a lower dichloromethane phase (with lipophilic compounds), with an intermediate phase of protein and cellular debris. The aqueous and organic phases were collected and evaporated to dryness. Chloroform was replaced by dichloromethane for security reason [46]. For the dietary intervention study, liver samples (100–120 mg) were homogenized in 1.2 mL methanol in a Fastprep-24 homogenizer (MP Biomedicals, Irvine, CA, USA). A homogenate that corresponded to 50 mg of tissue was extracted as described above for NMR analysis, and two homogenates from the same sample that corresponded to 1 mg of tissue were extracted for GC and LC-MS analyses.

### 4.3. GC Analysis of Neutral Lipids and Fatty Acids 

To analyze neutral lipids, we introduced three internal standards (3 µg of stigmasterol, 3 µg of cholesteryl heptadecanoate, and 15 µg of glyceryl trinonadecanoate) before extracting lipids from the homogenates. The dichloromethane phases were evaporated to dryness and dissolved in 20 µL ethyl acetate. Then, 1 µL of the lipid extract was analyzed with GC on a FOCUS-FID system (Thermo Electron, Waltham, MA, USA) equipped with a Zebron-1 fused silica capillary column (Phenomenex, Torrance, CA, USA; 5 m × 0.32 mm i.d., 0.50 µm film thickness) [47]. The oven temperature was programmed to increase from 200 °C to 350 °C at a rate of 5 °C per min, and the carrier gas was hydrogen (0.5 bar). The injector and detector were maintained at 315 °C and 345 °C, respectively.

To analyze fatty acid methyl ester (FAME), we introduced the internal standard, glyceryl tri heptadecanoate (2 µg), before extracting lipids from the homogenates. The lipid extracts were hydrolyzed in KOH (0.5 M in methanol) at 50 °C for 30 min, and transmethylated in a 10% boron trifluoride methanol solution (1 mL, Sigma-Aldrich, St. Louis, MO, USA) and hexane (1 mL) at 80 °C for one hour. After adding water (1 mL) to the crude solution, FAMEs were extracted with hexane (3 mL), evaporated to dryness, and dissolved in ethyl acetate (20 µL). FAMEs (1 µL) were analyzed with gas-liquid chromatography [48] on a Clarus 600-FID system (Perkin Elmer, Waltham, MA, USA) equipped with a Famewax fused silica capillary column (RESTEK, Lisses, France; 30 m × 0.32 mm i.d. 0.25 µm film thickness). The oven temperature was programmed to increase from 130 °C to 220 °C at a rate of 2 °C per min, and the carrier gas was hydrogen (0.5 bar). The injector and the detector were maintained at 225 °C and 245 °C, respectively.

### 4.4. HPLC-MS Analysis of Phospholipids

Lipids were extracted from 1 mg of liver with a method adapted from that described by Bligh and Dyer [26]. Extractions were performed in dichloromethane/methanol (2% acetic acid)/water (2.5:2.5:2 *v/v/v*), in the presence of six internal standards, including ceramides (Cer, d18:1/15:0, 16 ng); phosphatidylethanolamine (PE 12:0/12:0, 180 ng); phosphatidylcholine (PC, 13:0/13:0, 16 ng); SM (d18:1/12:0, 16 ng); phosphatidylinositol (PI, 16:0/17:0, 30 ng); and phosphatidylserine (PS, 12:0/12:0, 156.25 ng). The solution was centrifuged at 1500 rpm for 3 min. The organic phase was collected and dried under nitrogen, then dissolved in 50 µl methanol. The extract (5 µL) was analyzed with an Agilent 1290 UPLC system coupled to a G6460 triple quadrupole spectrometer (Agilent Technologies, Santa Clara, CA, USA) and equipped with MassHunter software, for data acquisition and analysis. A Kinetex HILIC column (Phenomenex, 50 × 4.6 mm, 2.6 µm) was used for LC separations. The column temperature was controlled at 40 °C. The flow rate of the mobile phase was 0.3 mL/min. The mobile phase contained two parts: A was acetonitrile; and B was 10 mM ammonium formate in water, pH 3.2. The gradient was prepared with the following specifications: from 10% to 30% B in 10 min; 10–12 min in 100% B; and then, at 13 min, back to 10% B for 1-min re-equilibrium, prior to the next injection. An electrospray source was employed in positive ion mode for Cer, PE, PC, and SM analyses and in negative ion mode for PI and PS analyses. The collision gas was nitrogen. The needle voltage was set at +4000 V. Several scan modes were used. First, to obtain the natural masses of different species, we analyzed cellular lipid extracts with precursor ion scans of 184 m/z, 241 m/z, and 264 m/z for PC/SM, PI, and Cer, respectively; and neutral loss scans of 141 and 87 for PE and PS, respectively. The collision energy optimums for Cer, PE, PC, SM, PI, and PS were 25 eV, 20 eV, 30 eV, 25 eV, 45 eV, and 22 eV, respectively. Then, the corresponding SRM transitions were used to quantify different phospholipid species in each class. Two MRM acquisitions were necessary, due to important differences between phospholipid classes. Data were analyzed with QqQ Quantitative (vB.05.00) and Qualitative analysis software (vB.04.00).

### 4.5. ^1^H-NMR Measurements

All NMR experiments were performed on a Bruker Avance spectrometer (Bruker Biospin, Rheinstetten, Germany), operating at a proton frequency of 600.13 MHz, with an inverse detection 5-mm ^1^H-^13^C-^15^N cryoprobe attached to a Cryoplatform (the preamplifier unit).

Dry lipid extracts were reconstituted in 500 µl CDCl_3_/CD_3_OD (4:1, *v/v*) and transferred into 5-mm NMR tubes. ^1^H-NMR spectra were recorded in the presence of a reusable coaxial capillary tube that contained 120 µl TSP (1.17 mM) in D_2_O, which also served as an internal standard for quantitative estimations. Dry aqueous extracts were reconstituted in 600 µl D_2_O containing TSP (0.70 mM) and transferred into 5-mm NMR tubes. 

A T1 (spin-lattice relaxation) measurement experiment was performed on the entire liver lipid extract (with TSP solution in the coaxial capillary as reference) in a sealed tube using an inversion-recovery sequence. The T1 values for TC (0.68 ppm), FC (1.01 ppm), CE (1.02 ppm), TG (5.27 ppm), TG (4.16 and 4.32 ppm), PL (5.23 ppm), UFA (5.35 ppm), linoleic acid (2.76 ppm), PUFA (2.82 ppm), FA (0.88 ppm), w3 UFA (0.98 ppm), MUFA (2.02 ppm), ARA+EPA (2.10 ppm), DHA (2.38 ppm) and TSP were 0.792, 0.792, 0.792, 1.44, 0.576, 1.44, 2.88, 1.58, 1.73, 3.17, 3.17, 1.44, 1.44, 1.15 and 3.45 s, respectively. 

^1^H-NMR spectra of liver lipid extracts were obtained with a one-pulse sequence, with a spectral width of 10 ppm, and the time domain data had 32,000 data points. The flip angle of the radio-frequency pulse was 30°, and the total relaxation delay was 15 s to ensure complete recovery of the magnetization between scans of the lipid components and for the external reference TSP. For each sample, 256 scans were accumulated, and data were Fourier-transformed, after multiplying by an exponential window function with a line-broadening function of 0.3 Hz to the free induction decays (FIDs).

^1^H-NMR spectra of aqueous liver extracts were acquired at 300 K with a conventional presaturation pulse sequence for water suppression, based on the first increment of the nuclear Overhauser effect spectroscopy (NOESY) pulse sequence. Solvent presaturation was applied during a recycling delay and mixing time (100 ms) to suppress residual water. A total of 256 transients were collected into 32,000 data points with a spectral width of 12 ppm and a relaxation delay of 15 s. Prior to the Fourier transform procedure, we applied an exponential line-broadening of 0.3 Hz to the FIDs.

### 4.6. Data Processing and Multivariate Analysis

NMR spectra were phase- and baseline-corrected, then calibrated (TSP, 0.0 ppm for aqueous extracts and TC, 0.68 ppm for lipidic extracts) with Topspin software (version 2.1, Bruker). Next, NMR data were reduced with AMIX software (version 3.9, Bruker) to integrate 0.01 ppm-wide regions that corresponded to the δ 10.0–0.5 ppm and the δ 6.4–0.6 ppm regions for aqueous and lipidic extracts, respectively. The 5.1–4.5 ppm region, which included water resonance, was excluded in the NMR spectra of aqueous extracts. We included 757 and 552 NMR buckets in the data matrices for aqueous and lipidic extracts, respectively. To account for differences in sample volumes, each integration region was normalized to the total spectral area. 

Multivariate pattern-recognition techniques were applied to study the effects of diet on the metabolome. First, we performed a PCA to reveal intrinsic clusters and detect eventual outliers. We then performed a PLS-DA to model the relationship between diet and NMR data. For aqueous extracts, prior to PLS-DA modeling, we applied orthogonal signal correction [49] to remove confounding variability; i.e., variability that was not linked to the diet (e.g., physiological, experimental, or instrumental variability). Data were Pareto-scaled (the square root of the standard deviation is used as the scaling factor). For all plots of scores, we performed Hotelling’s T2 statistics to construct 95% confidence ellipses. The R^2^Y parameter represented the explained variance. Seven-fold cross validation was used to determine the number of latent variables that should be included in the PLS-DA model and to estimate the predictive ability (or predicted variance, Q^2^ parameter) of the fitted model. PLS-DA models with Q^2^ values higher than 0.4 were considered valid [50]. In addition, the robustness of PLS-DA models was assessed with a permutation test (number of permutations = 200). In the permutation plot, a Q^2^ intercept < 0.05 indicated a robust model [51]. Discriminant variables were determined with the Variable Importance in the Projection (VIP) value, a global measure of the influence of each variable in the PLS components. Variables with VIPs > 1 were considered discriminants. Finally, we tested the significance of relative integration differences between groups, based on the non-parametric Kruskal-Wallis test. The false discovery rate (FDR) was applied to account for multiple testing. NMR variables that showed FDR-adjusted *p*-values < 0.05 were considered significantly different. We used SIMCA-P software (V13, Umetrics, Umea, Sweden) to perform the multivariate analyses, and we used R (https://www.r-project.org/) for univariate testing. 

### 4.7. Statistical Analysis

We performed analytical validations of ^1^H-NMR lipid quantifications by comparing the results with analogous measurements obtained with other methods. Comparisons were performed with linear regression and Pearson’s (r) correlation.

Linear regression analyses were used to compare known concentrations of lipidic species and signal integrations obtained with ^1^H-NMR. A known concentration was used as the independent variable, and the NMR-predicted concentration was used as the response variable. Hypotheses of linear regression were assessed based on the residuals: the Durbin-Watson, Shapiro-Wilks, and Breush-Pagan tests were applied, respectively, to test for the independence, normality, and homogeneity of residuals.

Pearson’s correlation was used to compare LipSpin-computed and NMR-computed quantifications of lipids and to compare GC-FID-computed and NMR-computed quantifications. We set 0.05 as the threshold for significance. Univariate analyses were performed with R software (https://www.r-project.org/).

## 5. Conclusions

The current study showed the potential and limitations of ^1^H-NMR spectroscopy for quantifying aqueous, and specially, lipid metabolites in the liver. We demonstrated that ^1^H-NMR spectra could provide a rapid overview of the major lipid classes and the most abundant aqueous metabolites. To achieve better extractions of aqueous and lipid metabolites and more accurate quantifications, we recommend the Folch extraction method, an external standard, and the CDCl_3_/CD_3_OD mixture of solvents. We found that LipSpin was a good alternative for lipid quantification, but the parameters must be optimized. Our metabolomics analysis allowed us to discriminate between livers of mice fed a diet deficient in essential fatty acids from livers of mice fed a balanced diet. The COCO dietary challenge was mainly associated with disturbances in lipid and energy metabolism, accompanied by altered amino acid metabolism.

## Figures and Tables

**Table 1:** Pearson’s r correlations and regression slopes indicate the similarity between NMR quantifications and real lipid concentrations for lipid mixtures dissolved in the solvent, CDCl_3._

Lipid Species	External Standard (TSP)			Internal Standard (TMS)		
	Pearson’s r	*p*-value ^a^	Slope	Pearson’s r	*p*-value ^a^	Slope
Total FA	0.98	0.002	1.3	0.82	0.089	0.8
Saturated FA	0.98	0.002	1.2	0.97	0.006	1.0
ω-3 FA	0.98	0.002	1.0	0.94	0.019	1.8
MUFA	0.98	0.001	1.2	0.93	0.019	2.0
PUFA	0.92	0.03	0.8	0.95	0.015	1.7
UFA	0.94	0.017	0.8	0.95	0.014	1.7
DHA	0.99	0.0001	1.1	0.93	0.024	1.9
Linoleic acid	0.98	0.002	0.9	0.93	0.025	1.7
TC	0.99	0.0003	1.2	0.89	0.04	2.5
FC	0.95	0.011	1.0	0.89	0.04	1.7
CE	0.99	0.00007	0.8	0.98	0.004	1.4
Triglycerides	0.99	0.001	1.2	0.98	0.004	1.0
PC	0.95	0.012	1.2	0.96	0.009	1.2
PE	0.55	0.33	0.1	0.99	0.0001	0.2
SM	0.94	0.018	0.8	0.98	0.002	0.9
Total PL	0.91	0.03	0.9	0.95	0.015	1.0

^a^ p value of the Pearson test (H0: *r* = 0); Abbreviations: TSP, 3-(trimethylsilyl)propionic acid sodium salt; TMS, tetramethylsilane; FA, fatty acids; MUFA, monounsaturated fatty acids; PUFA, monounsaturated fatty acids; UFA, unsaturated fatty acids; DHA, docosahexaenoic acid; TC, total cholesterol; FC, free cholesterol; CE, cholesterol ester; PC, phosphatidylcholine; PE, phosphatidylethanolamine; SM, sphingomyelin; PL, phospholipids.

**Table 2:** Pearson’s r correlations and regression slopes indicate the similarity between NMR quantifications and real lipid concentrations for lipid mixtures dissolved in the mixture of solvents, CDCl_3_/CD_3_OD (4:1).

Lipid Species	External Standard (TSP)			Internal Standard (TMS)		
	Pearson’s r	*p*-value ^a^	Slope	Pearson’s r	*p*-value ^a^	Slope
Total FA	0.99	5 × 10^−5^	1.0	0.98	2 × 10^−3^	1.2
Saturated FA	0.99	2 × 10^−5^	1.0	0.98	2 × 10^−3^	1.2
ω-3 FA	0.99	5 × 10^−5^	1.0	0.98	2 × 10^−3^	1.0
MUFA	0.99	3 × 10^−5^	1.1	0.99	4 × 10^−4^	1.0
PUFA	0.99	5 × 10^−4^	1.0	0.97	6 × 10^−3^	1.5
UFA	0.99	3 × 10^−4^	1.0	0.97	5 × 10^−3^	1.4
DHA	0.99	1 × 10^−5^	1.1	0.99	4 × 10^−5^	0.7
Linoleic acid	0.99	8 × 10^−6^	0.9	0.99	2 × 10^−4^	1.0
TC	0.98	2 × 10^−3^	1.1	0.96	9 × 10^−3^	1.6
FC	0.99	6 × 10^−4^	1.0	0.93	2 × 10^−2^	1.5
CE	0.99	8 × 10^−4^	0.9	0.98	2 × 10^−3^	0.9
Triglycerides	0.99	3 × 10^−6^	1.0	0.99	7 × 10^−4^	1.0
PC	0.99	5 × 10^−5^	1.2	0.99	2 × 10^−4^	1.1
PE	0.98	3 × 10^−3^	0.6	0.91	3 × 10^−2^	0.5
SM	0.99	8 × 10^−4^	0.9	0.96	8 × 10^−3^	0.8
Total PL	0.99	3 × 10^−5^	1.0	0.99	2 × 10^−5^	1.2

^a^ pvalue of the Pearson test (H0: *r* = 0); Abbreviations: TSP, 3-(trimethylsilyl)propionic acid sodium salt; TMS, tetramethylsilane; FA, fatty acids; MUFA, monounsaturated fatty acids; PUFA, monounsaturated fatty acids; UFA, unsaturated fatty acids; DHA, docosahexaenoic acid; TC, total cholesterol; FC, free cholesterol; CE, cholesterol ester; PC, phosphatidylcholine; PE, phosphatidylethanolamine; SM, sphingomyelin; PL, phospholipids.

**Table 3:** Pearson’s r correlations indicate the similarity between NMR and GC-FID quantifications of lipid concentrations in livers of mice in the dietary study.

Lipid Species	Pearson’s r	*p*-value ^a^
Total FA	0.93	8.8 × 10^−8^
Saturated FA	0.85	1.43 × 10^−5^
ω-3 FA	0.80	1.1 × 10^−4^
MUFA	0.96	4.5 × 10^−10^
PUFA	0.80	9.6 × 10^−5^
ARA+EPA	0.69	2 × 10^−3^
DHA	0.95	3.4 × 10^−9^
Linoleic acid	0.96	6.7 × 10^−10^
MUFA/PUFA	0.89	1.3 × 10^−6^
Total cholesterol	0.99	1.2 × 10^−14^
Free cholesterol	0.91	1.3 × 10^−6^
Cholesterol ester	0.98	6.6 × 10^−12^
Triglycerides	0.98	7.7 × 10^−12^

^a^ p value of the Pearson test (H0: *r* = 0); Abbreviations: FA, fatty acids; MUFA, monounsaturated fatty acids; PUFA, polyunsaturated fatty acids; DHA, docosahexaenoic acid.

**Table 4:** Comparison between ^1^H-NMR and LC-MS methods for determining ratios of phospholipid concentrations in livers from mice fed different diets in the dietary study.

Concentration Ratio	PE		PC + LPC		SM		Total PL, Except LPC	
	LC-MS	NMR	LC-MS	NMR	LC-MS	NMR	LC-MS	NMR
COCO/REF	0.94	0.95	1.07	1.01	0.90	0.81	1.05	1.03
FISH/REF	1.24	1.17	1.13	1.06	1.00	1.10	1.12	1.08

Abbreviations: COCO, a diet with 5% saturated FA-rich oil; REF: a diet with 5% reference oil (control); FISH: a diet with 5% n-3 long-chain PUFA-rich oil; PE, phosphatidylethanolamine; PC, phosphatidylcholine; LPC, lysophosphatidylcholine; PL, phospholipids;.

**Table 5:** Pearson’s r correlations between LipSpin and NMR quantifications of lipid concentrations in livers from mice fed different diets in the dietary study.

Lipid Species	Pearson’s r	*p*-value ^a^
Saturated FA	0.66	5.7 × 10^−3^
ω-3 FA	0.98	7.4 × 10^−12^
MUFA	0.97	3.0 × 10^−10^
ARA+EPA	0.001	0.99
DHA	0.31	0.24
Linoleic acid	0.95	2.8 × 10^−8^
Free cholesterol	0.54	0.031
Esterified cholesterol	0.80	1.8 × 10^−4^
Triglycerides	0.97	9.2 × 10^−10^
Total phospholipids	0.27	0.32
PE	0.58	0.017
SM	0.14	0.59
PC+LPC	0.37	0.15

^a^ p value of the Pearson test (H0: *r* = 0); Abbreviations: FA, fatty acids; MUFA, monounsaturated fatty acids; ARA, arachidonic acid; EPA, eicosapentaenoic acid; DHA, docosahexaenoic acid; PE, phosphatidylethanolamine; SM, sphingomyelin; PC, phosphatidylcholine; LPC, lysophosphatidylcholine.

**Table 6:** Fold-change of discriminants metabolites from binning data in mouse liver extracts induced by the COCO and FISH diets compared to the REF diet.

Metabolites ^a^	FC ^b^ COCO	FC ^b^ FISH
FA (CH_2_)_n_	0.71 *	0.82
EPA+ARA	0.67 *	0.80
FA CH3	0.73 *	0.80
Linoleic acid	0.28 *	0,92
MUFA	1.99 *	0.84
PC+LPC+SM	0.61 *	0.94
PL (Except LPC)	0.65 *	1.15
TG	1.63 *	0.98
3-Hydroxybutyrate	1.21 *	1.10
Alanine	1.30 *	0.97
Choline	1.02	1.68 *
Glucose	0.88 *	0.88 *
Glutamine	1	1.18 *
Glutathione	1.07	1.30 *
GPC	1.39 *	1.61 *
Inosine	1.24 *	1
Lactate	1.27 *	1.14 *
Leucine	1.23 *	1.11 *
Phenylalanine	1.32 *	1.15
Succinate	1.50 *	1.38 *
Threonine	1.28 *	1.03
Tyrosine	1.47 *	1.28 *
Valine	↑1.21 *	1.07

^a^ Metabolites that were significantly different between groups; relative integrations of buckets were compared between groups with the Kruskal-Wallis test and a multiple test correction (*p* < 0.05). ^b^FC, Fold Change = test diet/control diet. * indicates a significative difference between diets. Abbreviations: COCO, a diet with 5% saturated FA-rich oil; REF, a diet with 5% reference oil; FISH, a diet with 5% n-3 long-chain PUFA-rich oil; FA, fatty acids; EPA, eicosapentaenoic acid; ARA, arachidonic acid; MUFA, monounsaturated FAs; PC, phosphatidylcholine; LPC, lysophosphatidylcholine; SM, sphingomyelin; PL, phospholipids; TG, triglycerides; GPC, glycerophosphocholine.

## Supplementary Materials

The following are available online at https://www.mdpi.com/2218-1989/10/1/9/s1, Table S1: Compounds identified in the polar extract of mouse liver tissue from NMR data: assigned chemical shifts and multiplicities, Table S2: Compounds identified in the non-polar extract of mouse liver tissue from NMR data: assigned chemical shifts and multiplicities, Table S3: Composition of lipid mixtures, Table S4: real lipid concentrations and concentrations computed from NMR data for lipid mixtures of Table S3, Table S5: lipid concentrations computed from GC and NMR data in mice livers, Table S6: NMR bucketing table for aqueous liver samples, Table S7: NMR bucketing table for lipidic liver samples, Figure S1: 600 MHz ^1^H-NMR spectrum of a mixture of five lipid standards (TG C17:0; FC; oleate cholesterol; DHA; linoleic acid), Figure S2: 600 MHz ^1^H-NMR spectrum of a mixture of three lipid standards (PC, PE, SM), Figure S3: Scatter plot and linear regressions of NMR quantifications and lipid concentrations in lipid mixtures of Table S3 dissolved in CDCl_3_ and using an external standard (TSP) for neutral lipids and phospholipids, Figure S4: Scatter plot and linear regressions of NMR quantifications and lipid concentrations in lipid mixtures of Table S3 dissolved in CDCl3 and using an external standard (TSP) for fatty acids, Figure S5: Scatter plot and linear regressions of NMR quantifications and lipid concentrations in lipid mixtures of Table S3 dissolved in CDCl_3_ and using an internal standard (TMS) for neutral lipids and phospholipids, Figure S6: Scatter plot and linear regressions of NMR quantifications and lipid concentrations in lipid mixtures of Table S3 dissolved in CDCl_3_ and using an internal standard (TMS) for fatty acids, Figure S7: Scatter plot and linear regressions of NMR quantifications and lipid concentrations in lipid mixtures of Table S3 dissolved in CDCl_3_-CD_3_OD mixture and using an external standard (TSP) for neutral lipids and phospholipids, Figure S8: Scatter plot and linear regressions of NMR quantifications and lipid concentrations in lipid mixtures of Table S3 dissolved in CDCl_3_-CD_3_OD mixture and using an external standard (TSP) for fatty acids, Figure S9: Scatter plot and linear regressions of NMR quantifications and lipid concentrations in lipid mixtures of Table S1 dissolved in CDCl_3_-CD_3_OD mixture and using an internal standard (TMS) for neutral lipids and phospholipids, Figure S10: Scatter plot and linear regressions of NMR quantifications and lipid concentrations in lipid mixtures of Table S1 dissolved in CDCl_3_-CD_3_OD mixture and using an internal standard (TMS) for neutral lipids and phospholipids.

## References

[1] Friedman S.L., Neuschwander-Tetri B.A., Rinella M., Sanyal A.J. (2018). Mechanisms of NAFLD development and therapeutic strategies. Nat. Med..

[2] Younossi Z., Tacke F., Arrese M., Chander Sharma B., Mostafa I., Bugianesi E., Wai-Sun Wong V., Yilmaz Y., George J., Fan J. (2019). Global Perspectives on Nonalcoholic Fatty Liver Disease and Nonalcoholic Steatohepatitis. Hepatology.

[3] Dumas M.-E., Kinross J., Nicholson J.K. (2014). Metabolic phenotyping and systems biology approaches to understanding metabolic syndrome and fatty liver disease. Gastroenterology.

[4] Mardinoglu A., Bjornson E., Zhang C., Klevstig M., Söderlund S., Ståhlman M., Adiels M., Hakkarainen A., Lundbom N., Kilicarslan M. (2017). Personal model-assisted identification of NAD+ and glutathione metabolism as intervention target in NAFLD. Mol. Syst. Biol..

[5] Emwas A.-H., Roy R., McKay R.T., Tenori L., Saccenti E., Gowda G.A.N., Raftery D., Alahmari F., Jaremko L., Jaremko M. (2019). NMR Spectroscopy for Metabolomics Research. Metabolites.

[6] Le Moyec L., Triba M.N., Nahon P., Bouchemal N., Hantz E., Goossens C., Amathieu R., Savarin P. (2017). Nuclear magnetic resonance metabolomics and human liver diseases: The principles and evidence associated with protein and carbohydrate metabolism. Biomed. Rep..

[7] Amathieu R., Triba M.N., Goossens C., Bouchemal N., Nahon P., Savarin P., Le Moyec L. (2016). Nuclear magnetic resonance based metabolomics and liver diseases: Recent advances and future clinical applications. World J. Gastroenterol..

[8] Jiang L., Si Z.H., Li M.H., Zhao H., Fu Y.H., Xing Y.X., Hong W., Ruan L.Y., Li P.M., Wang J.S. (2017). 1H NMR-based metabolomics study of liver damage induced by ginkgolic acid (15:1) in mice. J. Pharm. Biomed. Anal..

[9] Dagla I., Benaki D., Baira E., Lemonakis N., Poudyal H., Brown L., Tsarbopoulos A., Skaltsounis A.L., Mikros E., Gikas E. (2018). Alteration in the liver metabolome of rats with metabolic syndrome after treatment with Hydroxytyrosol. A Mass Spectrometry and Nuclear Magnetic Resonance-based metabolomics study. Talanta.

[10] Bonvallot N., Canlet C., Blas-Y-Estrada F., Gautier R., Tremblay-Franco M., Chevolleau S., Cordier S., Cravedi J.P. (2018). Metabolome disruption of pregnant rats and their offspring resulting from repeated exposure to a pesticide mixture representative of environmental contamination in Brittany. PLoS ONE.

[11] Chen M., Zheng H., Xu M., Zhao L., Zhang Q., Song J., Zhao Z., Lu S., Weng Q., Wu X. (2019). Changes in hepatic metabolic profile during the evolution of STZ-induced diabetic rats via an 1H NMR-based metabonomic investigation. Biosci. Rep..

[12] Ghosh S., Sengupta A., Sharma S., Sonawat H.M. (2012). Metabolic fingerprints of serum, brain, and liver are distinct for mice with cerebral and noncerebral malaria: A ^1^H NMR spectroscopy-based metabonomic study. J. Proteome Res..

[13] Ruiz-Rodado V., Nicoli E.R., Probert F., Smith D.A., Morris L., Wassif C.A., Platt F.M., Grootveld M. (2016). 1H NMR-Linked Metabolomics Analysis of Liver from a Mouse Model of NP-C1 Disease. J. Proteome Res..

[14] Zheng H., Cai A., Shu Q., Niu Y., Xu P., Li C., Lin L., Gao H. (2019). Tissue-Specific Metabolomics Analysis Identifies the Liver as a Major Organ of Metabolic Disorders in Amyloid Precursor Protein/Presenilin 1 Mice of Alzheimer’s Disease. J. Proteome Res..

[15] Fernando H., Bhopale K.K., Kondraganti S., Kaphalia B.S., Ansari G.A.S. (2011). Lipidomic Changes in Rat Liver after Long-Term Exposure to Ethanol. Toxicol. Appl. Pharmacol..

[16] Fernando H., Bhopale K.K., Kondraganti S.S., Kaphalia B.S., Ansari G.A.S. (2018). Alcohol-Induced Hepatic Steatosis: A Comparative Study to Identify Possible Indicator(s) of Alcoholic Fatty Liver Disease. J. Drug Alcohol Res..

[17] Fernando H., Kondraganti S., Bhopale K.K., Volk D.E., Neerathilingam M., Kaphalia B.S., Luxon B.A., Boor P.J., Ansari G.A.S. (2010). 1H and 31P NMR Lipidome of Ethanol-Induced Fatty Liver. Alcohol. Clin. Exp. Res..

[18] Cabaton N.J., Poupin N., Canlet C., Tremblay-Franco M., Audebert M., Cravedi J.P., Riu A., Jourdan F., Zalko D. (2018). An Untargeted Metabolomics Approach to Investigate the Metabolic Modulations of HepG2 Cells Exposed to Low Doses of Bisphenol A and 17β-Estradiol. Front. Endocrinol..

[19] Wei F., Lamichhane S., Orešič M., Hyötyläinen T. (2019). Lipidomes in health and disease: Analytical strategies and considerations. TrAC Trends Anal. Chem..

[20] Khoury S., Canlet C., Lacroix M.Z., Berdeaux O., Jouhet J., Bertrand-Michel J. (2018). Quantification of Lipids: Model, Reality, and Compromise. Biomolecules.

[21] Martínez-Granados B., Morales J.M., Rodrigo J.M., Del Olmo J., Serra M.A., Ferrández A., Celda B., Monleón D. (2011). Metabolic profile of chronic liver disease by NMR spectroscopy of human biopsies. Int. J. Mol. Med..

[22] Cobbold J.F.L., Anstee Q.M., Goldin R.D., Williams H.R.T., Matthews H.C., North B.V., Absalom N., Thomas H.C., Thursz M.R., Cox R.D. (2009). Phenotyping murine models of non-alcoholic fatty liver disease through metabolic profiling of intact liver tissue. Clin. Sci. Lond. Engl..

[23] Teilhet C., Morvan D., Joubert-Zakeyh J., Biesse A.S., Pereira B., Massoulier S., Dechelotte P., Pezet D., Buc E., Lamblin G. (2017). Specificities of Human Hepatocellular Carcinoma Developed on Non-Alcoholic Fatty Liver Disease in Absence of Cirrhosis Revealed by Tissue Extracts ^1^H-NMR Spectroscopy. Metabolites.

[24] Vinaixa M., Rodríguez M.A., Rull A., Beltrán R., Bladé C., Brezmes J., Cañellas N., Joven J., Correig X. (2010). Metabolomic assessment of the effect of dietary cholesterol in the progressive development of fatty liver disease. J. Proteome Res..

[25] Lin C.Y., Wu H., Tjeerdema R.S., Viant M.R. (2007). Evaluation of metabolite extraction strategies from tissue samples using NMR metabolomics. Metabolomics.

[26] Bligh E.G., Dyer W.J. (1959). A rapid method of total lipid extraction and purification. Can. J. Biochem. Physiol..

[27] Folch J., Lees M., Sloane Stanley G.H. (1957). A simple method for the isolation and purification of total lipides from animal tissues. J. Biol. Chem..

[28] Beckonert O., Keun H.C., Ebbels T.M.D., Bundy J., Holmes E., Lindon J.C., Nicholson J.K. (2007). Metabolic profiling, metabolomic and metabonomic procedures for NMR spectroscopy of urine, plasma, serum and tissue extracts. Nat. Protoc..

[29] Ulrich E.L., Akutsu H., Doreleijers J.F., Harano Y., Ioannidis Y.E., Lin J., Livny M., Mading S., Maziuk D., Miller Z. (2008). BioMagResBank. Nucleic Acids Res..

[30] Wishart D.S., Tzur D., Knox C., Eisner R., Guo A.C., Young N., Cheng D., Jewell K., Arndt D., Sawhney S. (2007). HMDB: The Human Metabolome Database. Nucleic Acids Res..

[31] Reis A., Rudnitskaya A., Blackburn G.J., Mohd Fauzi N., Pitt A.R., Spickett C.M. (2013). A comparison of five lipid extraction solvent systems for lipidomic studies of human LDL. J. Lipid Res..

[32] Wu H., Southam A.D., Hines A., Viant M.R. (2008). High-throughput tissue extraction protocol for NMR-and MS-based metabolomics. Anal. Biochem..

[33] Viant M.R., Ebbels T.M.D., Beger R.D., Ekman D.R., Epps D.J.T., Kamp H., Leonards P.E.G., Loizou G.D., MacRae J.I., van Ravenzwaay B. (2019). Use cases, best practice and reporting standards for metabolomics in regulatory toxicology. Nat. Commun..

[34] Bharti S.K., Roy R. (2012). Quantitative 1H NMR spectroscopy. TrAC Trends Anal. Chem..

[35] Vidal N.P., Manzanos M.J., Goicoechea E., Guillén M.D. (2012). Quality of farmed and wild sea bass lipids studied by (1) H NMR: Usefulness of this technique for differentiation on a qualitative and a quantitative basis. Food Chem..

[36] Barrilero R., Gil M., Amigó N., Dias C.B., Wood L.G., Garg M.L., Ribalta J., Heras M., Vinaixa M., Correig X. (2018). LipSpin: A New Bioinformatics Tool for Quantitative 1H NMR Lipid Profiling. Anal. Chem..

[37] Ducheix S., Montagner A., Polizzi A., Lasserre F., Marmugi A., Bertrand-Michel J., Podechard N., Al Saati T., Chétiveaux M., Baron S. (2013). Essential fatty acids deficiency promotes lipogenic gene expression and hepatic steatosis through the liver X receptor. J. Hepatol..

[38] Jiang C., Yang K., Yang L., Miao Z., Wang Y., Zhu H. (2013). A 1H NMR-Based Metabonomic Investigation of Time-Related Metabolic Trajectories of the Plasma, Urine and Liver Extracts of Hyperlipidemic Hamsters. PLoS ONE.

[39] Li J., Vosegaard T., Guo Z. (2017). Applications of nuclear magnetic resonance in lipid analyses: An emerging powerful tool for lipidomics studies. Prog. Lipid Res..

[40] Botolin D., Wang Y., Christian B., Jump D.B. (2006). Docosahexaneoic acid (22:6, n-3) regulates rat hepatocyte SREBP-1 nuclear abundance by Erk-and 26S proteasome-dependent pathways. J. Lipid Res..

[41] Jump D.B. (2011). Fatty acid regulation of hepatic lipid metabolism. Curr. Opin. Clin. Nutr. Metab. Care.

[42] Dentin R., Benhamed F., Pégorier J.P., Foufelle F., Viollet B., Vaulont S., Girard J., Postic C. (2005). Polyunsaturated fatty acids suppress glycolytic and lipogenic genes through the inhibition of ChREBP nuclear protein translocation. J. Clin. Investig..

[43] Klein M.S., Dorn C., Saugspier M., Hellerbrand C., Oefner P.J., Gronwald W. (2011). Discrimination of steatosis and NASH in mice using nuclear magnetic resonance spectroscopy. Metabolomics.

[44] Yang Y., Li C., Nie X., Feng X., Chen W., Yue Y., Tang H., Deng F. (2007). Metabonomic studies of human hepatocellular carcinoma using high-resolution magic-angle spinning 1H NMR spectroscopy in conjunction with multivariate data analysis. J. Proteome Res..

[45] Schofield Z., Reed M.A., Newsome P.N., Adams D.H., Günther U.L., Lalor P.F. (2017). Changes in human hepatic metabolism in steatosis and cirrhosis. World J. Gastroenterol..

[46] Chen I.S., Shen C.S.J., Sheppard A.J. (1981). Comparison of methylene chloride and chloroform for the extraction of fats from food products. J. Am. Oil Chem. Soc..

[47] Barrans A., Collet X., Barbaras R., Jaspard B., Manent J., Vieu C., Chap H., Perret B. (1994). Hepatic lipase induces the formation of pre-beta 1 high density lipoprotein (HDL) from triacylglycerol-rich HDL2. A study comparing liver perfusion to in vitro incubation with lipases. J. Biol. Chem..

[48] Lillington J.M., Trafford D.J., Makin H.L. (1981). A rapid and simple method for the esterification of fatty acids and steroid carboxylic acids prior to gas-liquid chromatography. Clin. Chim. Acta Int. J. Clin. Chem..

[49] Wold S., Antti H., Lindgren F., Öhman J. (1998). Orthogonal signal correction of near-infrared spectra. Chemom. Intell. Lab. Syst..

[50] McCombie G., Browning L.M., Titman C.M., Song M., Shockcor J., Jebb S.A., Griffin J.L. (2009). omega-3 oil intake during weight loss in obese women results in remodelling of plasma triglyceride and fatty acids. Metab. Off. J. Metab. Soc..

[51] Lapins M., Eklund M., Spjuth O., Prusis P., Wikberg J.E. (2008). Proteochemometric modeling of HIV protease susceptibility. BMC Bioinform..

