# Supplementary material for: Proton NMR Enables the Absolute Quantification of Aqueous Metabolites and Lipid Classes in Unique Mouse Liver Samples
Source: Metabolites. 2019 Dec 21;10(1):9. doi: 10.3390/metabo10010009 (PMC7023327; doi:10.3390/metabo10010009)
Supplement: Supplementary file 1 [file metabolites-10-00009-s001.zip › TableS1.pdf]

**Table S1**. Compounds identified in the polar extract of mouse liver tissue from NMR data : assigned chemical shifts and multiplicities[a]

| Compound Name | $^1$H NMR Chemical Shift (ppm), Multiplicity, Coupling constants (Hz) |
|---|---|
| **3-Hydroxybutyrate** | **1.20** (d, J=6.4); 2.31 (dd, J=14.3 and 6.1); 2.41 (dd, J=14.3 and 7.6); 4.15 (m) |
| **Acetate** | **1.93** (s) |
| **Alanine** | **1.48** (d, J=7.2); 3.78 (q, J=7.2) |
| Aspartate | 2.68(dd, J=17.6 and 8.4); 2.82(dd, J=17.6 and 3.6); 3.90(m) |
| **Choline** | **3.21**(s); 3.53(m); 4.07(m) |
| **Creatine** | **3.04**(s); 3.93(s) |
| Ethanol | 1.19(t, J=7.1); 3.66(q, J=7.1) |
| **Formate** | **8.46**(s) |
| **Glucose** | 3.25(dd, J=8 and 9); 3.41(m); 3.48(m); **3.54**(dd, J=9.5 and 3.6); 3.72(m); 3.77(m); 3.84(m); **3.90**(dd, J=12.3 and 2.2); 4.65(d, J=7.8); 5.24(d, J=3.7) |
| **Glutamate** | 2.06(m); 2.12(m); **2.36**(m); 3.77(m) |
| **Glutamine** | 2.15(m); **2.46**(m); 3.78(m) |
| **Glutathione** | 2.18(m); **2.56(m)**; 2.97(m); 3.29(m); 3.78(m) |
| **Glycerol** | 3.56(dd, J=11.6 and 6.5); **3.66**(dd, J=11.6 and 4.3); 3.78(m) |
| Glycerophosphocholine | 3.23(s); 3.62(m); 3.68(m); 3.92(m); 4.33(m) |
| **Glycine** | **3.56**(s) |
| Inosine monophosphate | 4.02(m); 4.38(m); 4.51(m); 6.15(d, J=5.7); 8.24(s); 8.53(s) |
| **Inosine** | 4.28(m); 4.44(m); **6.11**(d, J=5.6); 8.24(s); 8.35(s) |
| **Isoleucine** | 0.94(t, J=6.8); **1.015**(d, J=6.8); 1.27(m); 1.47(m); 1.99(m); 3.68(d,J=4) |
| **Lactate** | 1.33(d, J=7); **4.11**(q, J=7) |
| **Leucine** | **0.965**(t, J=6.4); 1.72(m); 3.74(m) |
| **Methionine** | 2.12(m); 2.13(s); 2.20(m); **2.65**(t, J=7.6); 3.86(m) |
| **Niacinamide** | 8.94(d, J=2); 8.72(dd, J=5 and 2); 8.25(m); **7.60**(m) |
| **Phenylalanine** | 3.15(m); 3.29(m); 4.0(m); 7.33(d, J=7.6); 7.38(t, J=7.6); **7.43**(t, J=7.6) |
| **Succinate** | **2.42**(s) |
| **Taurine** | **3.27**(t, J=6.8); 3.43(t, J=6.8) |
| **Threonine** | 1.33(d, J=7);3.60(d, J=4.4); **4.26**(m) |
| **Tyrosine** | 3.07(m); 3.20(m); 3.95(m); **6.90**(d, J=8.4); 7.20(d, J=8.4) |
| **Uridine** | 4.23(t, J=5.2); 4.36(t, J=5.2); **5.91**(m); 7.88(d, J=8.2) |
| **Valine** | **0.99** (d, J=7); 1.05(d, J=7); 2.28(m); 3.62(d, J=4.2) |

[a]Metabolites quantified and signals used for quantifications are given in bold
