# Supplementary material for: Proton NMR Enables the Absolute Quantification of Aqueous Metabolites and Lipid Classes in Unique Mouse Liver Samples
Source: Metabolites. 2019 Dec 21;10(1):9. doi: 10.3390/metabo10010009 (PMC7023327; doi:10.3390/metabo10010009)
Supplement: Supplementary file 1 [file metabolites-10-00009-s001.zip › TableS2.pdf]

Table S2. Compounds identified in the non-polar extract of mouse liver tissue from NMR data : assigned chemical shifts and multiplicities[a]

**Compound Name**	**Assignment, ^{1}H NMR Chemical Shift (ppm), Multiplicity, Coupling constants (Hz)**
**Cholesterol ester**	C-18 H3 (0.68, s); **C-19 H3 (1.02,s)**
**Fatty acid chain**	CH3 SFA(0.88,t,J=6.5); CH3 w-6 (0.90, t, J=6.5); **CH3 w-3 (0.97, t, J=6.5)**; (CH2)n (1.2-1.4, m); CH2-C-CH=(1.75, m); CH2-CH=(1.98-2.09,m); CH2-CO(2.24-2.35,m); =CH-CH2-CH=(2.74-2.90,m); CH=CH(5.30-5.42, m)
**Free cholesterol**	C-18 H3(0.68,s); **C-19 H3 (1.01,s)**
**Glycerophospholipid** backbone	C-3 H2 (3.94,m); **C-2H (5.17-5.24, m)**
**Phosphatidylcholine**	**N(CH3)3 (3.20,s)**
**Phosphatidyl ethanolamine**	**N-CH2** (3.12,m)
**Sphingomyelin**	N(CH3)3 (3.23,s); **CH=CH (5.70,m)**
**Total cholesterol**	**C-18 H3 (0.68,s)**
**Triglycerides** (glycerol backbone)	C-1 H2/ C-3 H2 (4.15,dd, J=12 and 6 /**4.29,dd, 12 and 4.2**); C-2 H (5.27,m)

[a]Lipid classes quantified and signals used for lipid quantifications are given in bold
