# Supplementary material for: Proton NMR Enables the Absolute Quantification of Aqueous Metabolites and Lipid Classes in Unique Mouse Liver Samples
Source: Metabolites. 2019 Dec 21;10(1):9. doi: 10.3390/metabo10010009 (PMC7023327; doi:10.3390/metabo10010009)
Supplement: Supplementary file 1 [file metabolites-10-00009-s001.zip › TableS3.pdf]

Table S3. Composition of lipid mixtures

Compound	Concentration (mmol/l)									
	**Mix1**	**Mix2**	**Mix3**	**Mix4**	**Mix5**	**Mix6**	**Mix7**	**Mix8**	**Mix9**	**Mix10**
Cholesterol	0.62	0.77	0.45	0.36	0.15	---	---	---	---	---
Cholesterol oleate	0.35	0.47	0.20	0.16	0.07	---	---	---	---	---
Triglycerides (C17:0)	0.31	1.28	1.80	2.88	3.08	---	---	---	---	---
Linoleic acid	1.74	2.32	1.22	0.81	0.35	---	---	---	---	---
Docosahexaenoic acid	0.91	1.22	0.64	0.43	0.18	---	---	---	---	---
Phosphatidylcholine	---	---	---	---	---	0.68	0.85	1.02	1.36	1.53
Phosphatidyl-ethanolamine	---	---	---	---	---	0.15	0.12	0.10	0.07	0.05
Sphingomyelin	---	---	---	---	---	0.20	0.15	0.10	0.07	0.05
