# Supplementary figures and images for: Proton NMR Enables the Absolute Quantification of Aqueous Metabolites and Lipid Classes in Unique Mouse Liver Samples
Source: Metabolites. 2019 Dec 21;10(1):9. doi: 10.3390/metabo10010009 (PMC7023327; doi:10.3390/metabo10010009)

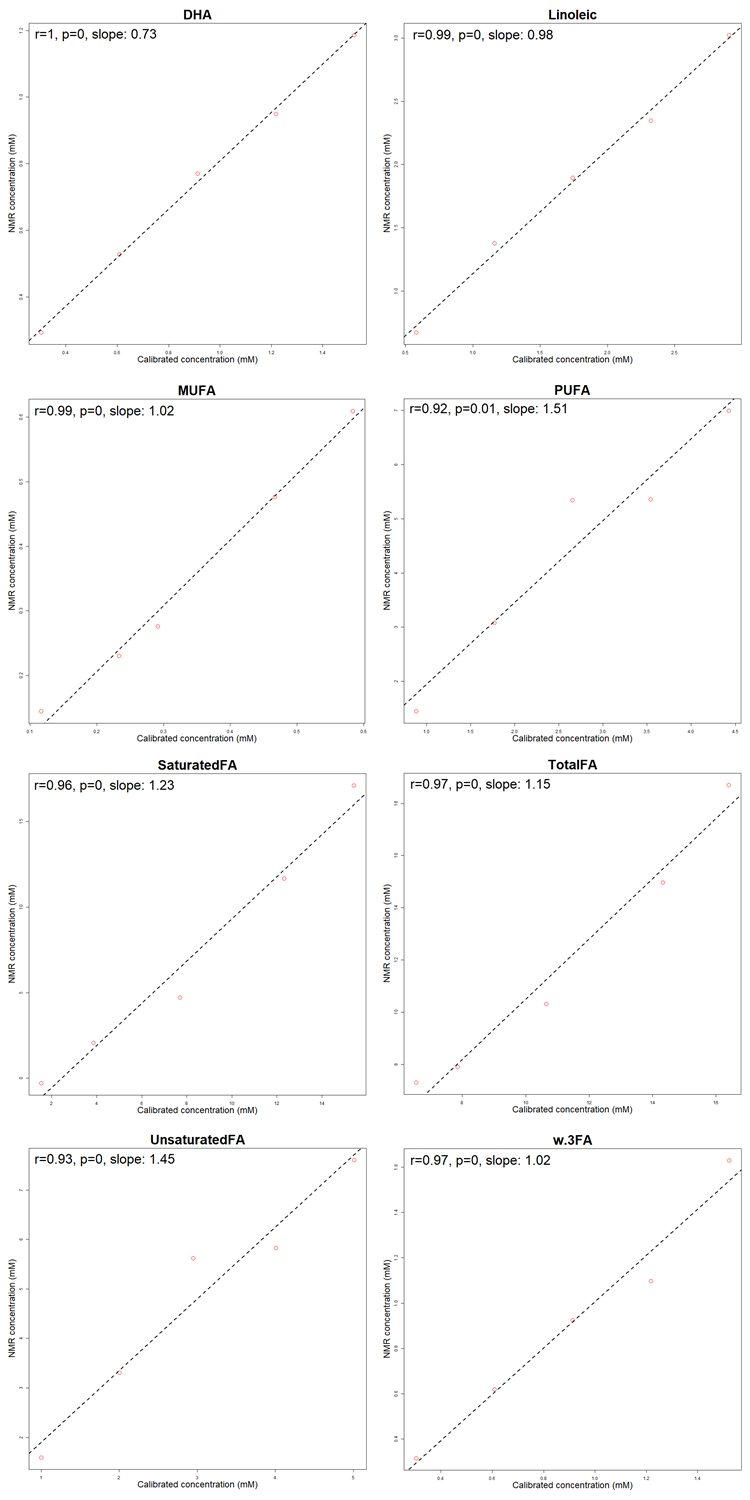

Supplement: Supplementary file 1 [file metabolites-10-00009-s001.zip › FigureS10modified.png]

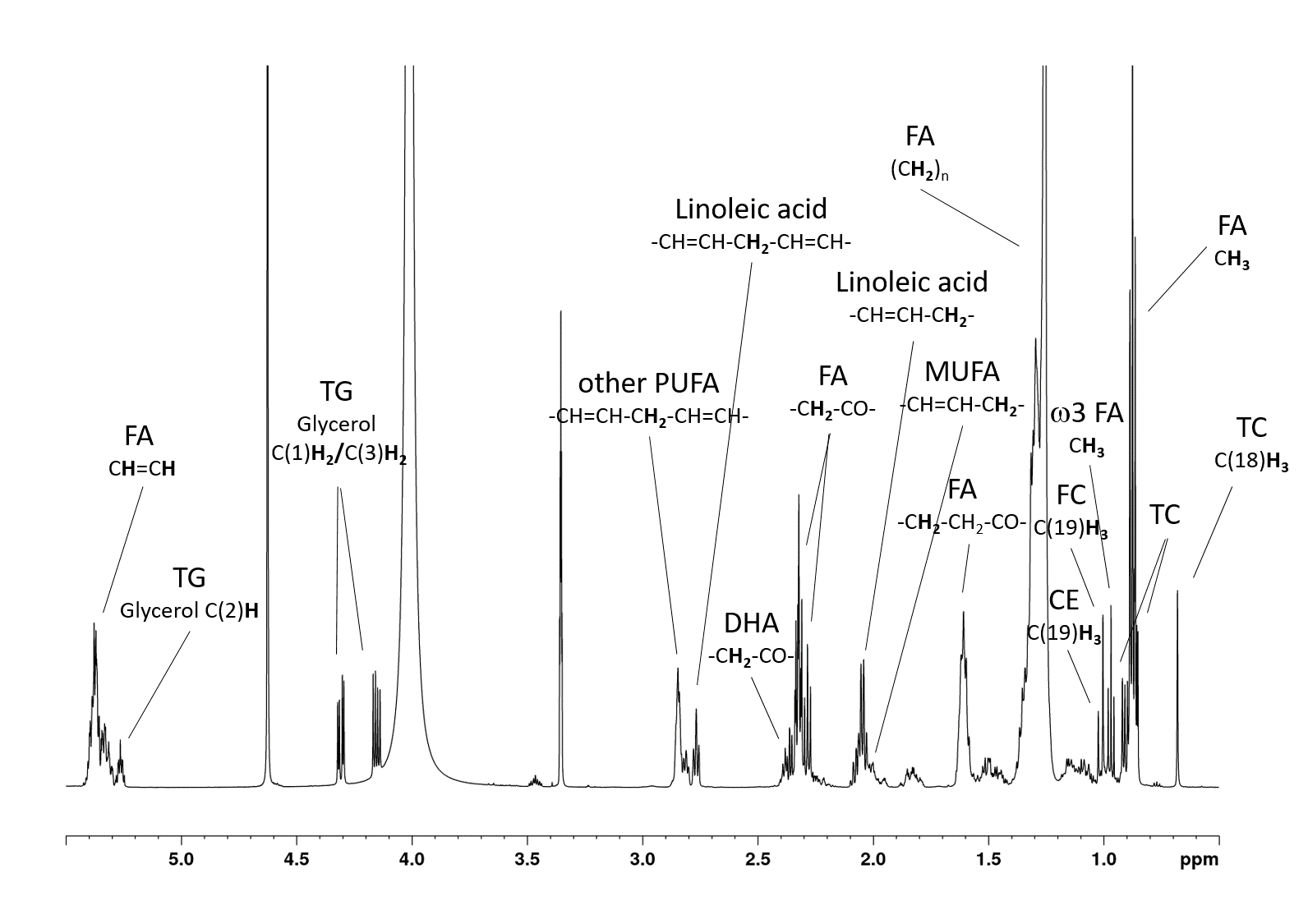

Supplement: Supplementary file 1 [file metabolites-10-00009-s001.zip › FigureS1modified.png]

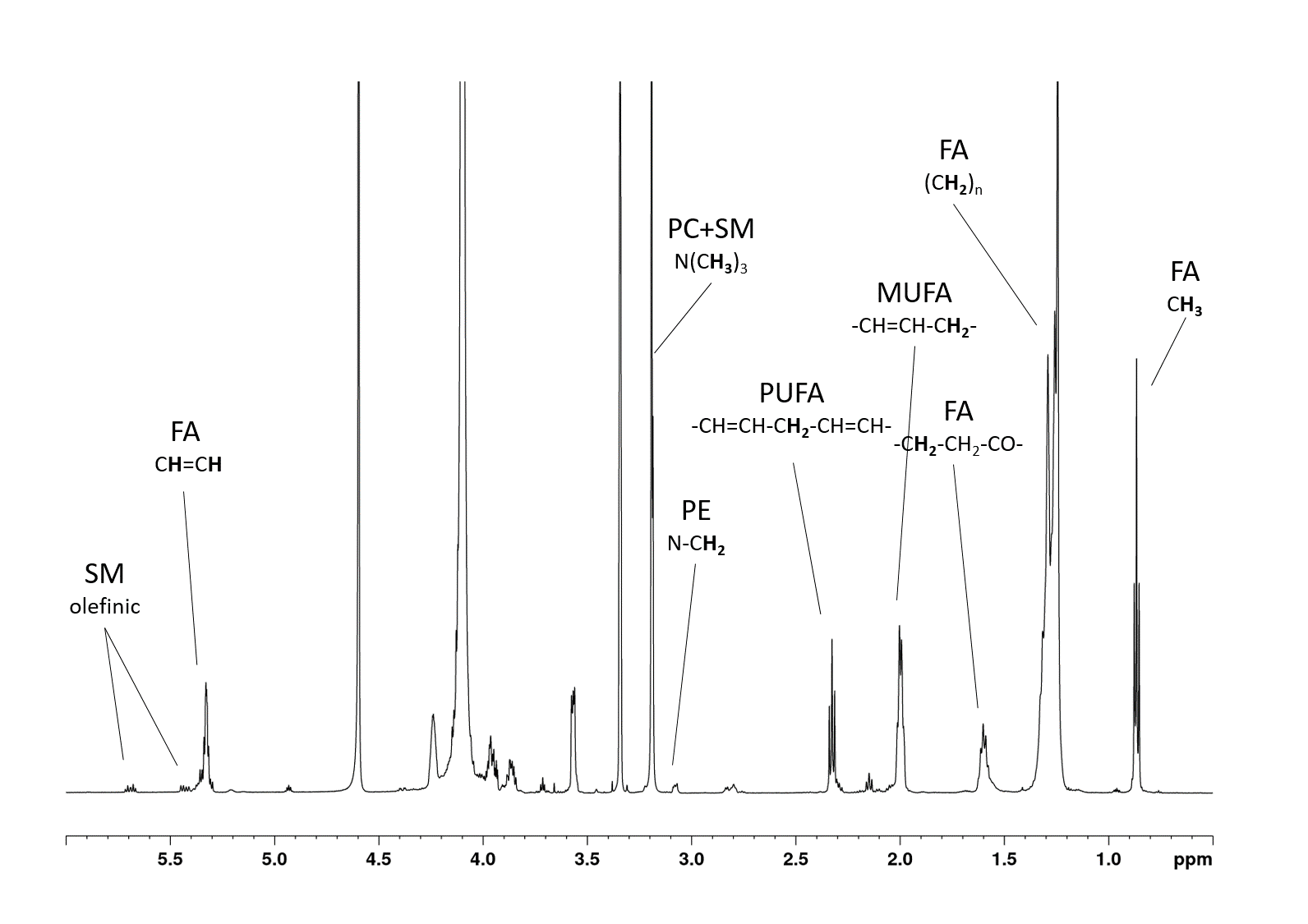

Supplement: Supplementary file 1 [file metabolites-10-00009-s001.zip › FigureS2modified.png]

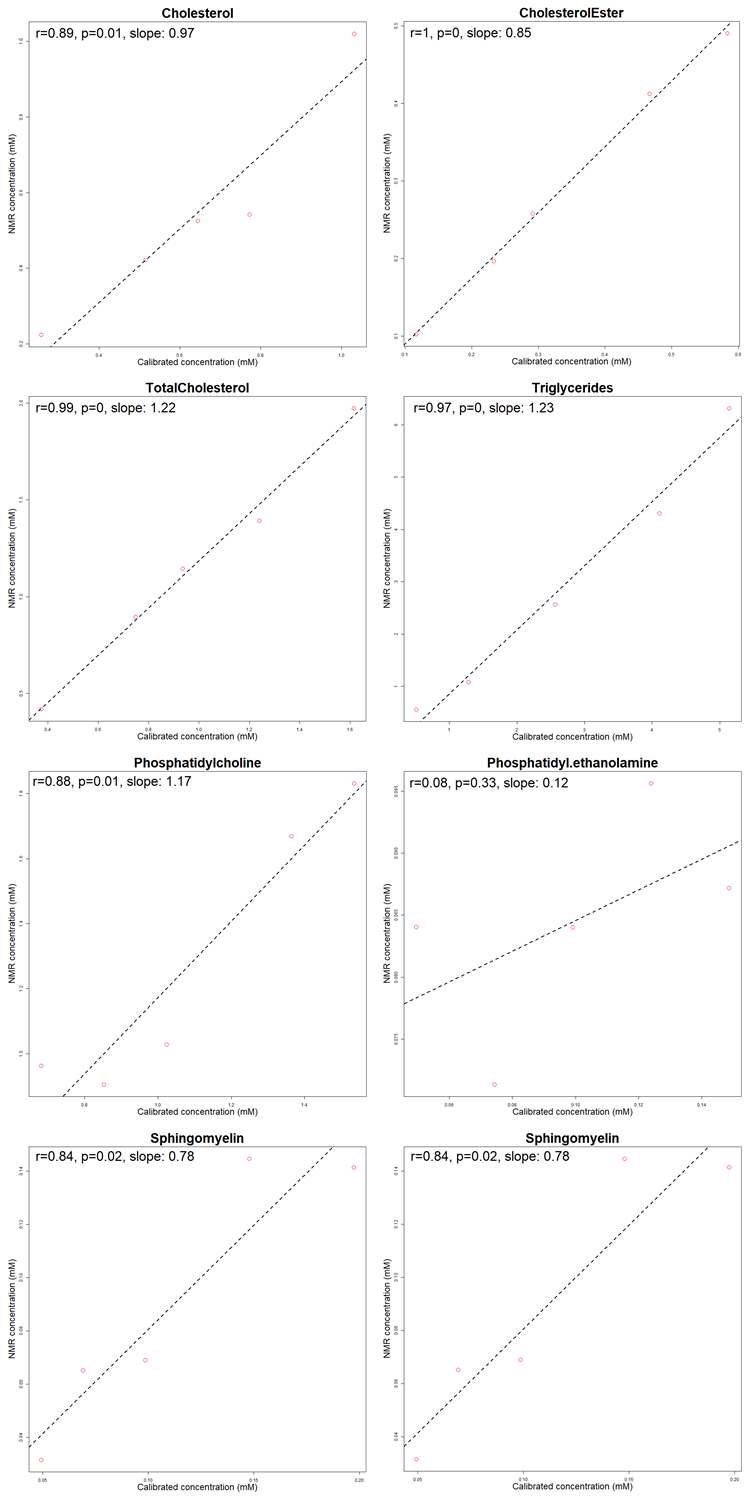

Supplement: Supplementary file 1 [file metabolites-10-00009-s001.zip › FigureS3modified.png]

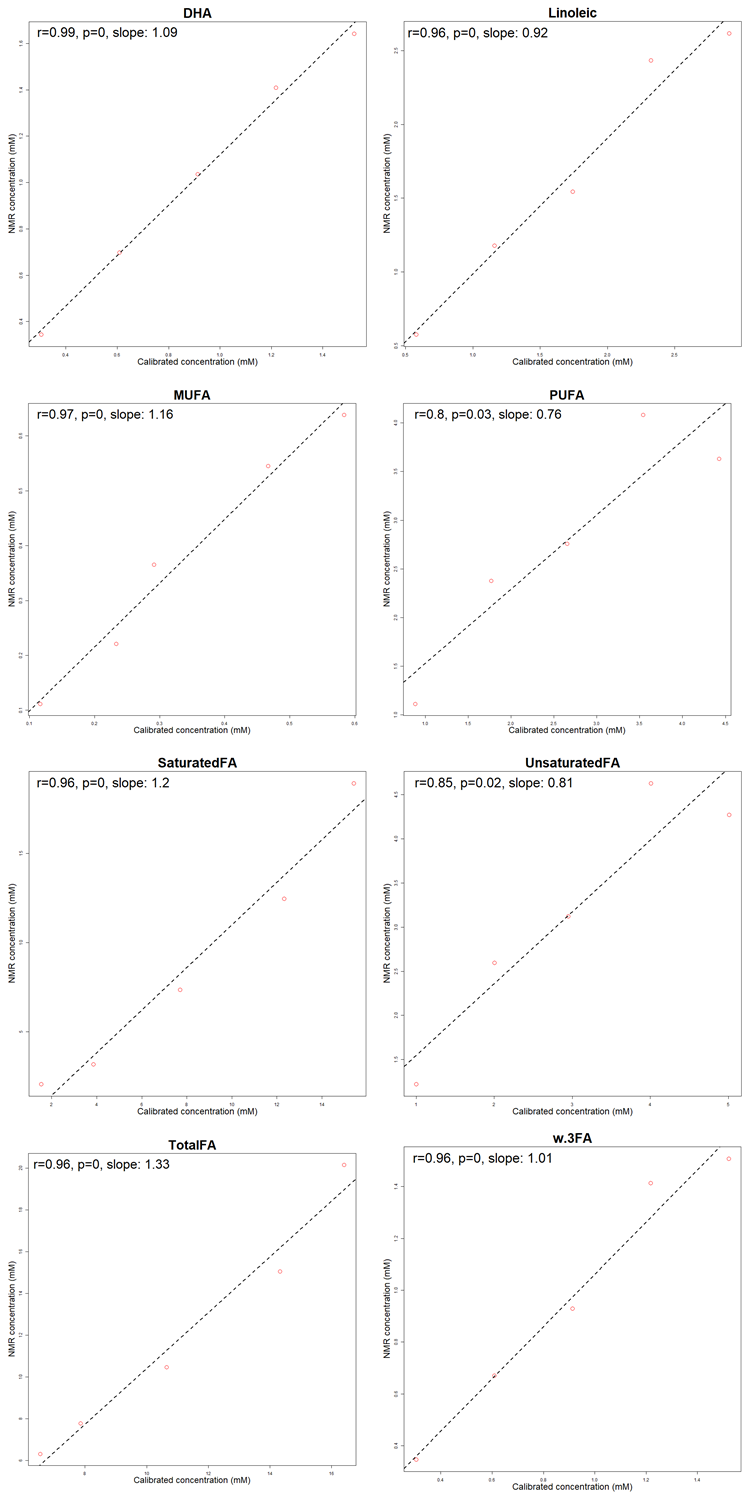

Supplement: Supplementary file 1 [file metabolites-10-00009-s001.zip › FigureS4modified.png]

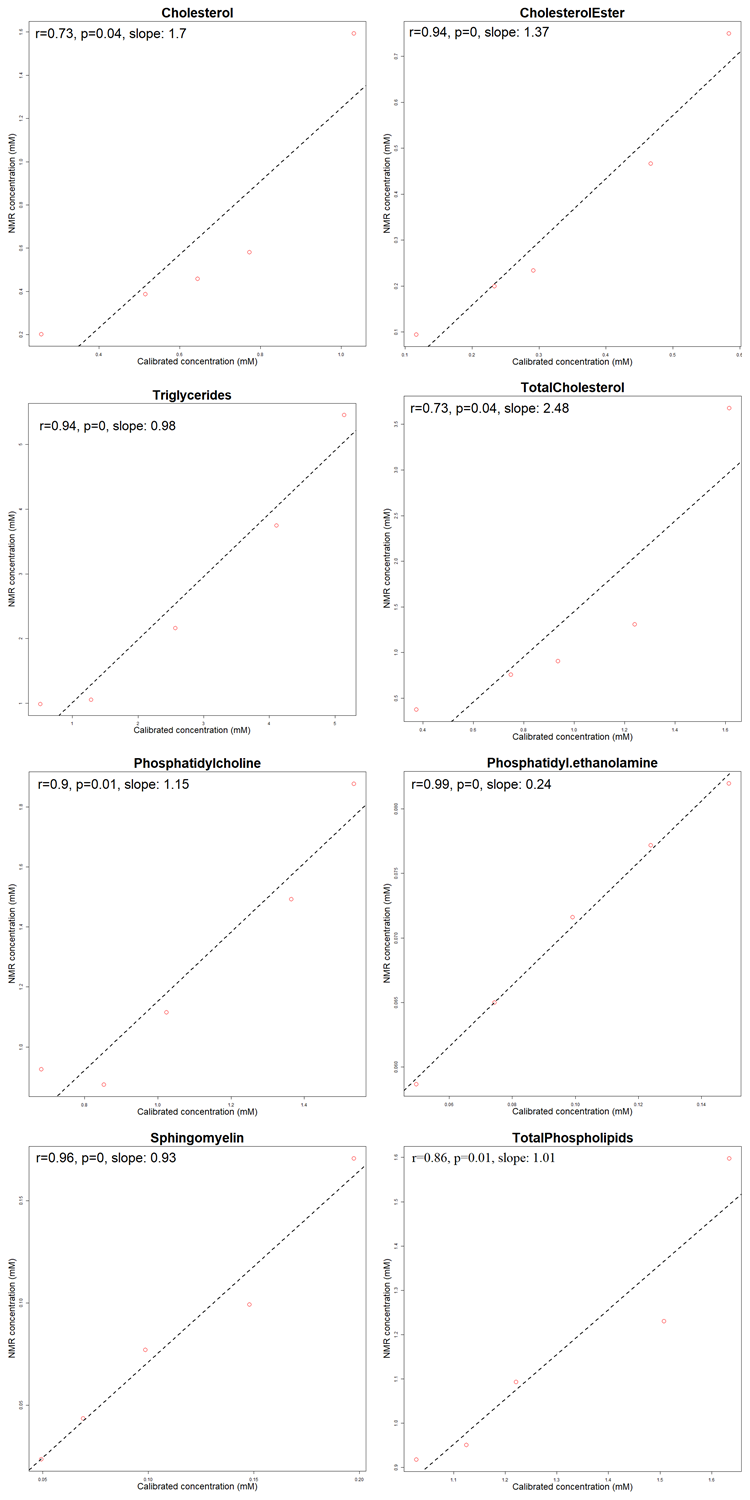

Supplement: Supplementary file 1 [file metabolites-10-00009-s001.zip › FigureS5modified.png]

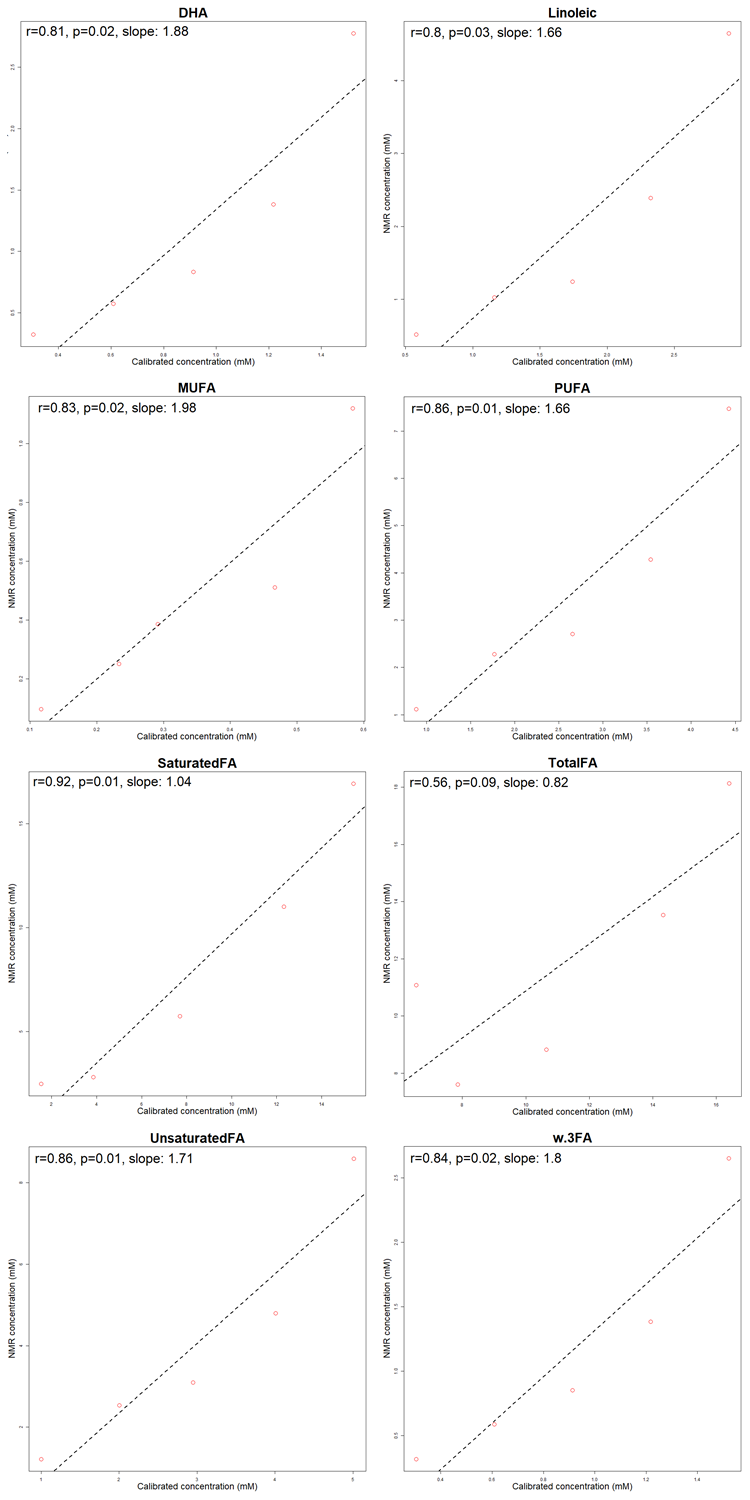

Supplement: Supplementary file 1 [file metabolites-10-00009-s001.zip › FigureS6modified.png]

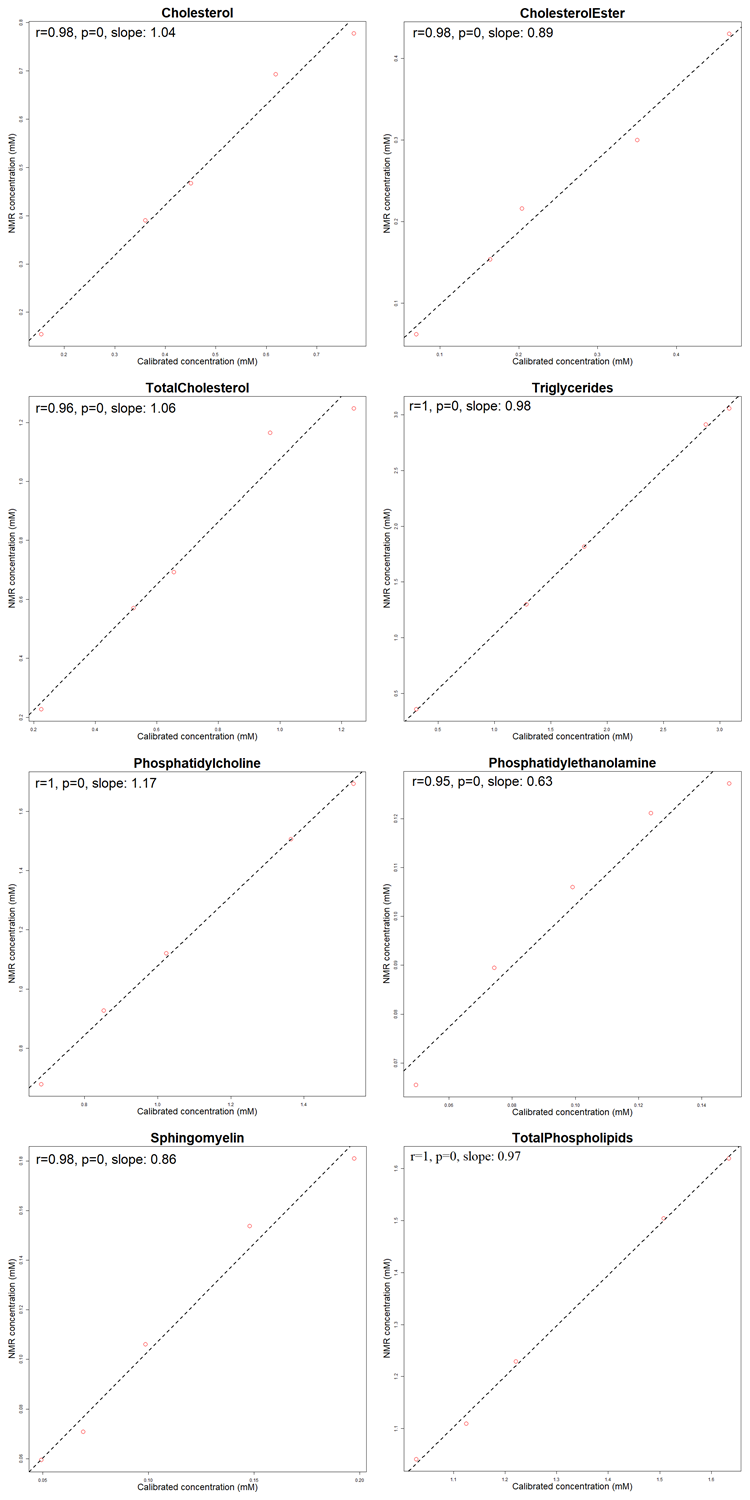

Supplement: Supplementary file 1 [file metabolites-10-00009-s001.zip › FigureS7modified.png]

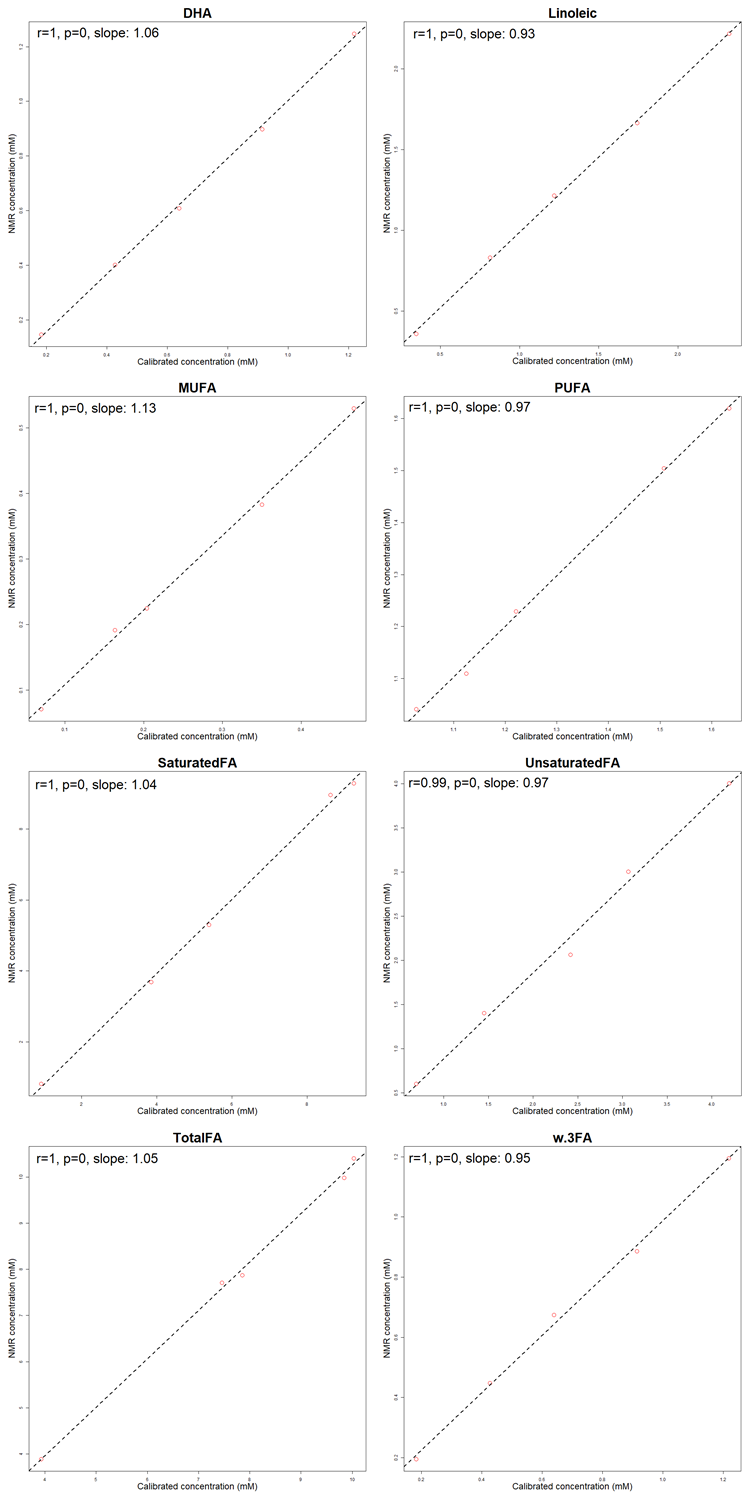

Supplement: Supplementary file 1 [file metabolites-10-00009-s001.zip › FigureS8modified.png]

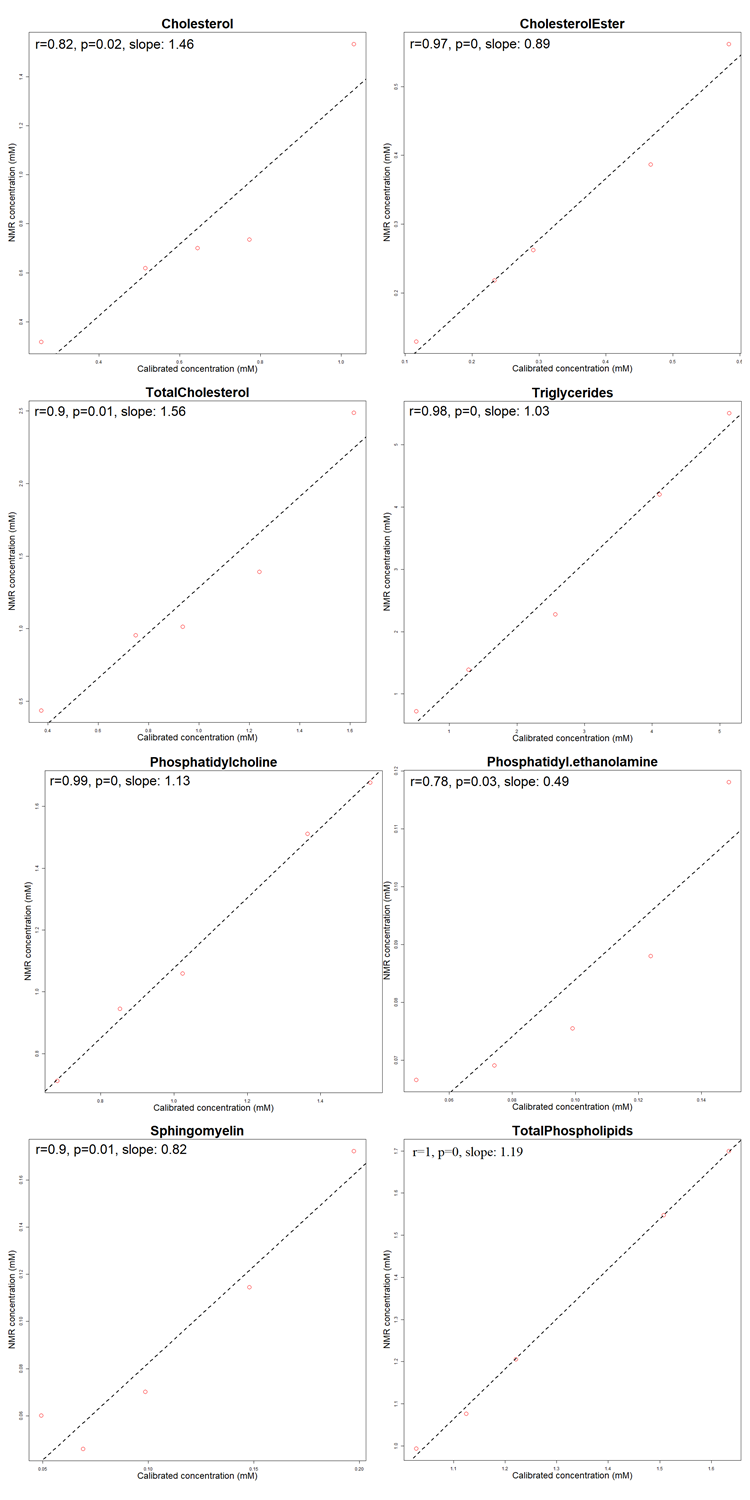

Supplement: Supplementary file 1 [file metabolites-10-00009-s001.zip › FigureS9modified.png]
